# Haloperidol induces neuroprotection and enhances neuromuscular function in both murine and human models of spinal muscular atrophy

**DOI:** 10.1038/s12276-026-01689-0

**Published:** 2026-04-13

**Authors:** Giovanna Menduti, Raquel Perez-Gomez, Noémie Berenger-Currias, Cristina Ruatti, Jorge Espinosa-Espinosa, Camille Januel, Piotr Konieczny, Ruben Artero, Cecile Martinat, Marina Boido

**Affiliations:** 1 Department of Neuroscience “Rita Levi Montalcini”, University of Turin, Turin, Italy; 2 Neuroscience Institute Cavalieri Ottolenghi, Orbassano, Turin, Italy; 3 Human Translational Genomics Group, University Institute of Biotechnology and Biomedicine, Universidad de Valencia, Burjassot, Spain; 4 Incliva Biomedical Research Institute, Valencia, Spain; 5 INSERM/UEVE, UMR 861, Université Paris Saclay, CECS/I-STEM, AFM-Telethon, Rue Henri Desbruères, Corbeil-Essonnes, France; 6 Centre for Biomedical Network Research on Rare Diseases (CIBERER), CB23/07/00005, Carlos III Health Institute, Madrid, Spain; 7 Experimental and Applied Biomedicine Research Group, Health Sciences Faculty, Universidad Particular Internacional SEK (UISEK), Quito, Ecuador

**Keywords:** Animal disease models, Bioinformatics, Drug delivery, Cell death in the nervous system, Neurodegeneration

## Abstract

Spinal muscular atrophy (SMA) is a severe neuromuscular disorder caused by *Survival Motor Neuron 1* (*SMN1*) gene mutations, leading to reduced SMN protein levels and progressive motor neuron (MN) degeneration. Although current therapies aim to restore SMN expression, limitations highlight the need for alternative strategies. We investigated haloperidol (HALO), a classical antipsychotic, as a potential therapeutic based on its ability to enhance *SMN2* splicing and SMN expression. Using the delta 7 SMA mouse model, we assessed effects of HALO on survival, motor function, neuroprotection, and neuroinflammation, by histological, molecular, and RNA-sequencing analyses of spinal cord and muscle samples. Additionally, we examined patient induced pluripotent stem cell-derived MNs and myotube co-cultures for validation in human cells. HALO increased lifespan and motor performance in mice with SMA, upregulated SMN protein in spinal cord and muscles, reduced MN loss, and attenuated neuroinflammation. Moreover, HALO enhanced neuromuscular junction integrity and muscle trophism, suggesting peripheral benefits. RNA-sequencing analysis revealed extensive splicing changes, including SMN target transcripts, supporting enhanced activity. In human models, HALO improved MN survival and SMN expression, supporting dual SMN-dependent and neuroprotective mechanisms. Given its central nervous system penetrance and clinical approval, HALO emerges as a promising SMA therapy candidate, warranting further dose optimization and validation for translational potential.

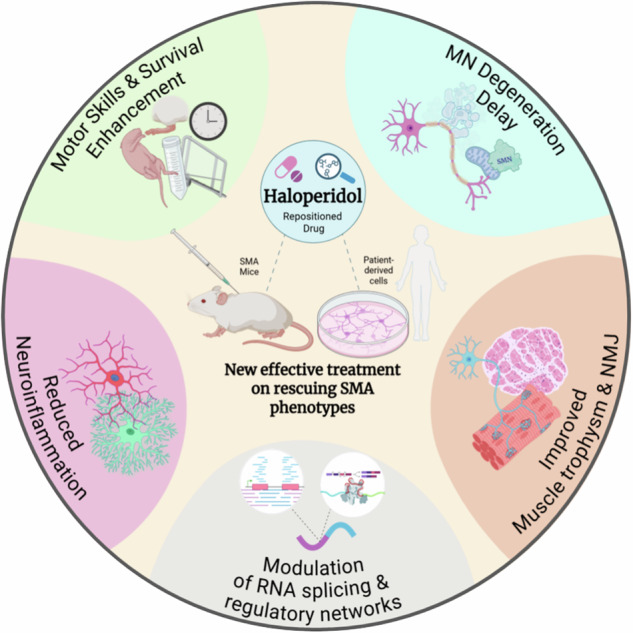

## Haloperidol shows promise as SMA treatment strategy

Spinal muscular atrophy (SMA) is a genetic disorder that affects muscle movement owing to a lack of a protein called survival motor neuron (SMN). Current treatments focus on increasing SMN levels but do not address all disease aspects. Researchers explored using haloperidol (HALO), an antipsychotic drug, to treat SMA by increasing SMN protein levels and correcting other SMA defects. Researchers administered HALO to mice and observed improvements in survival, weight, and motor skills. They also found that HALO increased SMN protein levels in the spinal cord and muscles, which are crucial for movement. In human cell tests, HALO improved cell survival and muscle function. Results suggest that HALO not only boosts SMN levels but also helps protect nerve cells and improve muscle health. This research indicates that HALO could be a promising treatment for SMA, potentially working alongside existing therapies.

This summary was initially drafted using artificial intelligence, then revised and fact-checked by the author.

## Introduction

Drug repositioning is a promising strategy to meet the critical therapeutic needs in rare diseases^1,2^ as it accelerates patient access by reducing time and costs while ensuring greater safety in clinical trials owing to the well-documented toxicology profile of the repurposed drug. In particular, this approach has gained significant attention in spinal muscular atrophy (SMA) research for its potential to also uncover novel and unexpected ways of addressing the disease^1,2^. SMA is a neuromuscular disease affecting children and young adults, characterized by the progressive loss of lower motor neurons (MNs) and muscle atrophy, caused by bi-allelic mutations in the *Survival Motor Neuron 1* (*SMN1*) gene. This leads to a deficiency of the SMN protein, essential for the survival and maintenance of MNs^3^. Consequently, all the currently available therapeutic therapies aim at increasing the levels of full-length SMN protein either by supplying a fully functioning copy of the human *SMN1* gene (Zolgensma™) or by increasing *SMN2* Ex7 inclusion (Nusinersen™ and Risdiplam™)^4^. Indeed, under SMA conditions, the loss of *SMN1* is partially compensated by its paralog *SMN2*, which, despite being unaffected, generates only about 10% of full-length SMN protein owing to alternative splicing, leading to exon 7 exclusion in ~90% of cases and producing a truncated SMN protein (Δ7-SMN)^5^. Although current therapeutic advances have substantially extended lifespan of patients and allowed treated children to reach unprecedented motor milestones, several fundamental challenges remain. These include administration methods, exclusion criteria, high costs, unknown long-term effects, and the predominant focus on SMN restoration^2^. Although SMN-targeted therapies enhance outcomes, they do not fully address all aspects of the disease. Notably, neuromuscular junction (NMJ) impairments persist in adults with SMA even after 14 months of nusinersen treatment, with no significant improvement in NMJ function or its correlation with physical performance^6^. These findings highlight the need for different therapies that address not only SMN deficits but also other disease pathways. Furthermore, strategies should target not only the increase of the SMN protein in MNs but also in other neural cells, including glial cells involved in SMA-related neuroinflammation^7–9^, as well as peripheral tissues such as muscles^10,11^, whose dysfunction precedes or even exacerbates MN degeneration and SMA symptoms.

Exploiting the drug repositioning approach for SMA, we developed a *Drosophila*-based reporter system that was informative of the human *SMN2* exon 7 inclusion in fly MNs and used it for in vivo drug screening of FDA-approved drugs^12^. The most promising drugs were validated in patient-derived cells (SMA II fibroblasts), and among these, moxifloxacin and haloperidol (HALO) (referred as “compound 5” in^12^; PCT/EP2022/087022) modulated the splicing of *SMN2* transcripts by promoting *SMN2* Ex7 inclusion. We previously demonstrated that moxifloxacin can be repositioned for the SMA treatment, as assessed both in human cell culture and mouse models^13^. Here, we explored the therapeutic potential of HALO, a well-established antipsychotic, highlighted in our screening for its ability to increase *SMN2* transcript expression in both isoforms, with and without Ex7^12^. HALO readily crosses the blood–brain barrier and exerts its clinical effects primarily through dopamine D2 receptor (DRD2) antagonism^14,15^. This antidopaminergic action underlies its widespread use in the treatment of schizophrenia, acute psychosis, Tourette syndrome, and other severe behavioral disorders as well as its investigation for potential modulating effect on the inflammasome^16,17^. On the basis of this evidence, here we report our investigations to confirm SMN-enhancing effects of HALO at optimized doses while minimizing its antipsychotic impact, both in vivo in delta 7 mice, a murine model of severe SMA^18^, and in vitro in patient induced pluripotent stem cell (iPSC)-derived MNs and primary SMA myoblasts.

## Materials and methods

### Study design

The identification of HALO as SMN enhancer was highlighted in our previous FDA-approved drug screening (in the patent PCT/EP2022/087022 for its use in SMA referred as “compound 5”)^12^, thus requiring further validation in different SMA models. We used both in vivo (mice with severe SMA) and in vitro models [human induced pluripotent stem cell (hiPSC) lines and immortalized myoblasts derived from SMA-affected patients]. In vivo, we administered HALO (0.5 mg/kg/day) or vehicle (VHL, 2% dimethyl sulfoxide in 0.9% NaCl solution) to delta 7 mice^18^, for 10 days (postnatal day (P)2–P12) or until survival, according to humane end points (that is, 20% loss of body weight relative to its maximum attained weight, inability of the animal to right itself, and evident respiratory distress). We evaluated survival, behavioral motor functions, and weight^19,20^, neuroprotection, neuroinflammation, and muscle trophism/innervation by performing histological (Nissl and hematoxylin/eosin stainings), immunofluorescence and molecular (immunoblotting) analyses on spinal cord, brain, and muscle (quadriceps and gastrocnemius) samples. In parallel, RNA-sequencing (RNA-seq) analysis was performed in spinal cord and muscle samples of treated mice to shed further light on molecular pathways targeted by HALO treatment. We also examined the effects of HALO in patient iPSC-derived MNs and myotube co-cultures to validate its therapeutic potential in human SMA cells. Specifically, we evaluated MN survival and neuritic networking, as well as myotube fusion area and the number of acetylcholine receptor clusters in co-cultures (immunofluorescence analysis), and assessed SMN protein levels (immunoblotting).

All experiments were conducted and analyzed randomly and blindly. Age-matched mice of both sexes were assigned to different treatment groups, with blinded data analysis. Eighty-five delta 7 mice with SMA were used for behavioral, histological, molecular, and survival analyses. Moreover, 14 wild-type (WT) mice were used for weight analysis, immunofluorescence, and/or western blot analysis. An a priori power analysis has been conducted with the G*Power software, based on the expected variability from our previous publications in which we used the same mouse strain and experimental techniques^13,21–23^. All experiments followed the ARRIVE guidelines for animal research and were conducted a minimum of three times. The results were consistently replicated in individual mice and cell cultures in each trial; all data meeting the technical validity criteria — including perfusion, tissue preparation, staining, recordings, and imaging — were included in the analysis.

The experimental procedures involving live animals were performed in strict accordance with the European Communities Council Directive 86/609/EEC Italian Ministry of Health and University of Turin institutional guidelines on animal welfare (law 116/92 on Care and Protection of living animals undergoing experimental or other scientific procedures; permit no. 980/20). In addition, the ad hoc Ethical Committee of the University of Turin specifically approved this study. For the in vitro studies on hiPSC lines, informed consent was obtained from all the patients included in this study, complying with the ethical guidelines of the institutions and with the legislation requirements. Experimental protocols were approved by the French Ministry of Health (2019-A02599-48).

### Statistical analysis

All the statistical details of the experiments are reported in figure legends, including the statistical tests used, the *n* meaning, and exact value. Unless otherwise stated, statistical analyses were performed using GraphPad Prism 8.0 software (GraphPad software, San Diego, CA, USA) and data are expressed as mean ± standard error of the mean (SEM). Survival analysis between HALO-injected and VHL-injected mice with SMA was performed using the Kaplan–Meier test (censoring mice sacrificed at P12), with log rank (Mantel–Cox) as a post hoc test. Weight and motor behavior data were analyzed using a mixed-effects model with Geisser–Greenhouse correction. The *P*-value for the column factor, representing the statistical difference among treated delta 7 mice groups, was reported. Post hoc comparisons were performed using Sidak’s multiple comparison test to assess differences between treatment groups of mice with SMA over time. Non-normally distributed data (righting reflex and negative geotaxis) were analyzed with Fisher’s exact test. Body weight data were presented as a Kaplan–Meier plot, showing the time from P2 until the postnatal day when an animal’s weight fell two standard deviations below the WT average, remaining below this threshold through P12. Unpaired two-tailed *t*-tests compared normally distributed two-group data; one-way analysis of variance with Tukey’s post hoc for more than two groups. Kruskal–Wallis, followed by Dunn’s multiple comparisons test, was applied for non-normally distributed data. The Benjamini–Hochberg method controlled the false discovery rate for RNA-seq analyses, with an adjusted *P*-value <0.05. Finally, values of *P* < 0.05 were considered statistically significant (*P < 0.05; **P < 0.005; ***P < 0.0005; ****P < 0.0001).

Supplementary materials and methods for this manuscript are available on the Experimental & Molecular Medicine website (http://www.nature.com/emm/) as part of the Supplementary materials, including sections on animal models, HALO/VHL administration, behavioral tests, tissue processing, immunoblotting, histology, hiPSC-derived MNs, co-cultures, treatments, immunostaining, RNA sequencing, LLM declaration, Supplementary Table 1 of antibodies, and the references for Supplementary materials and methods.

## Results

### HALO treatment improves weight, survival, and motor performance in delta 7 mice with SMA

To assess the efficacy of HALO administration in the delta 7 SMA mouse model^18^, we first evaluated whether the treatment (0.5 mg/kg/day) could extend the lifespan of the treated mice, mitigate SMA-related phenotypic signs, and improve their overall well-being. Notably, HALO-treated mice exhibited improved well-being and activity, with no observable adverse behavioral or sedation-related changes.

**Fig. 1:**
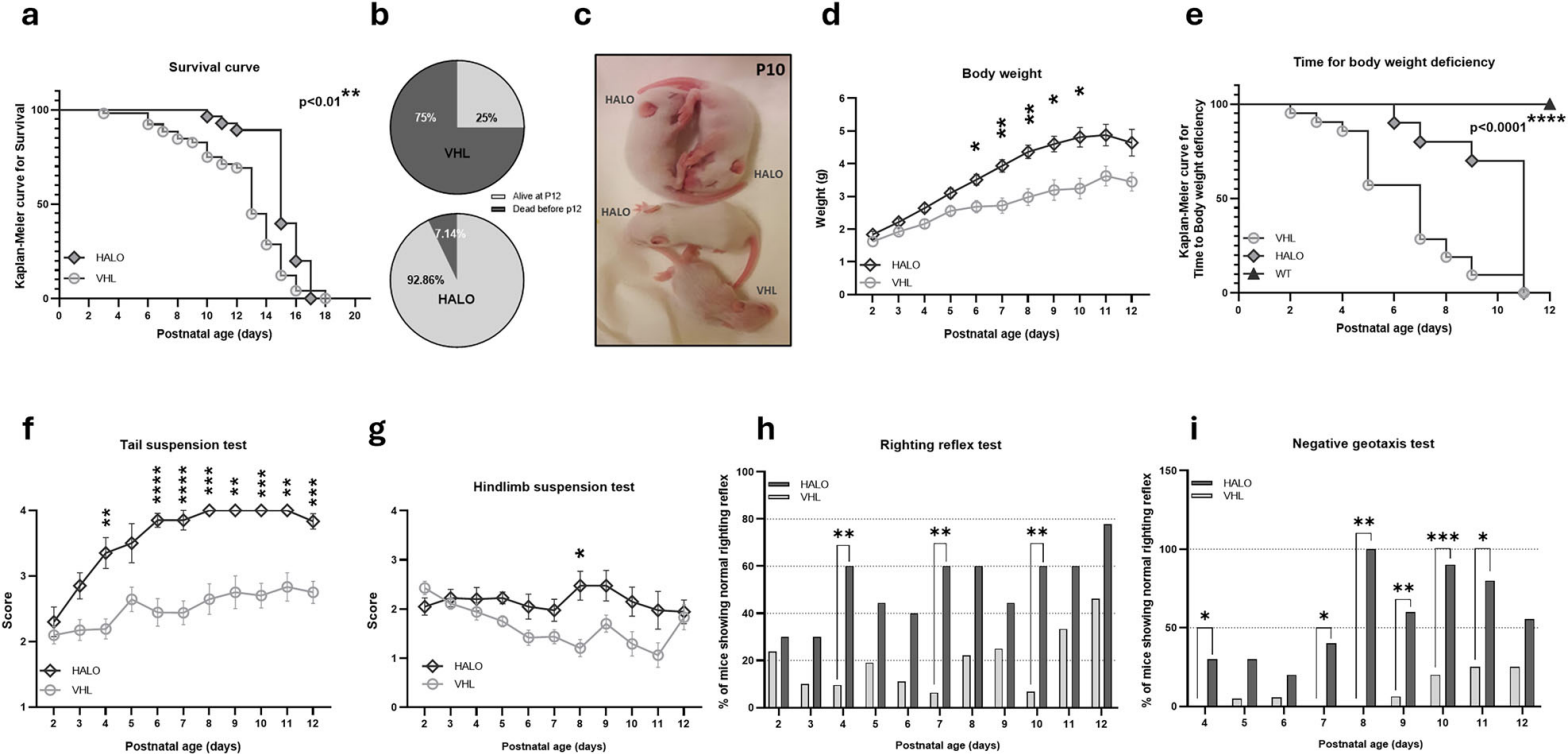
HALO treatment improves survival, body mass, and motor function in delta 7 mice with SMA. **a** Kaplan–Meier survival curve (censoring the mice sacrificed at P12) shows the significant haloperidol (HALO) effect on improving the lifespan of delta 7 mice (VHL *n* = 52, HALO *n* = 28). **b** Pie charts showing percentages of vehicle (VHL) and HALO mice survived to P12 (VHL *n* = 52, HALO *n* = 28). **c** Representative picture of HALO and VHL spinal muscular atrophy mice in the late stage of the disease (P10). **d** Body weight assessment from P2 to P12. **e** Event time plot for mice body weight deviation, VHL *n* = 21, HALO *n* = 10, wild-type (WT) *n* = 8. Tail suspension test (part **f**) and hindlimb suspension score of treated delta 7 mice (part **g**). In parts **d**, **f**, and **g**, data are expressed as mean ± SEM, VHL *n* = 21, HALO *n* = 10. Successful righting reflex (part **h**) and negative geotaxis of treated delta 7 mice (part **i**). Statistical analyses included Kaplan–Meier survival estimates (log-rank Mantel–Cox and Gehan–Breslow–Wilcoxon post hoc tests) (parts **a** and **e**), mixed-effects models (Geisser–Greenhouse correction, Sidak’s multiple comparisons) (parts **d**, **f**, and **g**), and Fisher’s exact test (parts **h** and **i**). All values are presented as mean ± SEM. Significance levels: *P < 0.05, **P < 0.01, ***P < 0.005, ****P < 0.0001. SEM, standard error of the mean.

By analyzing mice survival, we found that HALO administration significantly increased the lifespan of treated mice (+15.4%) compared with VHL group (Fig. 1a; median survival HALO = 15 days, VHL = 13 days; VHL *n* = 52, HALO *n* = 28). We also evaluated the percentage of mice with SMA that reached the age of P12, compared with those that died earlier: only 7.14% of HALO-treated pups died before P12 versus 25% of VHL mice (Fig. 1b).

To assess the treatment effect on motor skills of mice with SMA, HALO-treated and VHL-treated pups underwent weight assessment and behavioral tests. HALO-treated mice demonstrated an overall increase in the mean body weight compared with the VHL group (VHL *n* = 21, HALO *n* = 10) (Fig. 1c,d). Statistical analysis showed significant treatment (*F*(1,29) = 18.04, ***P < 0.001), time (*F*(2.370,58.77) = 57.31, ****P < 0.0001), and treatment–time interaction effects (*F*(10,248) = 7.436, ****P < 0.0001). Furthermore, HALO treatment significantly delayed body weight loss in comparison to VHL mice, highlighting different onsets of SMA worsening compared with WT controls (VHL *n* = 21, HALO *n* = 10, WT *n* = 8) (Fig. 1e).

The behavioral results revealed a general improvement in hindlimb posture and muscle strength in HALO-treated mice with SMA compared with controls (for all the behavioral motor tests: VHL *n* = 21, HALO *n* = 10). HALO mice exhibited nearly fully spread hindlimbs in comparison with VHL (with hindlimbs often closed together), both in the tail suspension test (Fig. 1f; by P4 and from P6 to P11) and in the hindlimb suspension test (Fig. 1g; with a onefold increase at P8). Regarding the evaluation of motor development (reflexes) and muscle weakness, the analysis of righting reflex (Fig. 1h) and negative geotaxis tests (Fig. 1i) highlighted significant improvements in HALO mice, as already evident at P4 and further confirmed from P7 to P11. In addition, the latency to complete the tests (Supplementary Fig. 1a,b) revealed a statistically significant difference between the groups both in righting time at P7, P8, and P10 and in negative geotaxis at P10. This additional measure complements test completion rates by indicating that, although some VHL mice eventually completed the tasks at late disease stages, their longer latencies reflect motor deficits and underscore the improved performance in HALO-treated mice.

Overall, these results suggest a general improvement in the survival, health (weight gain), strength, and motor coordination (behavioral tests) of mice with SMA upon HALO administration compared with VHL animals.

### HALO exerts a neuroprotective effect and delays MN impairment in both mice with SMA and patient-derived MNs

**Fig. 2:**
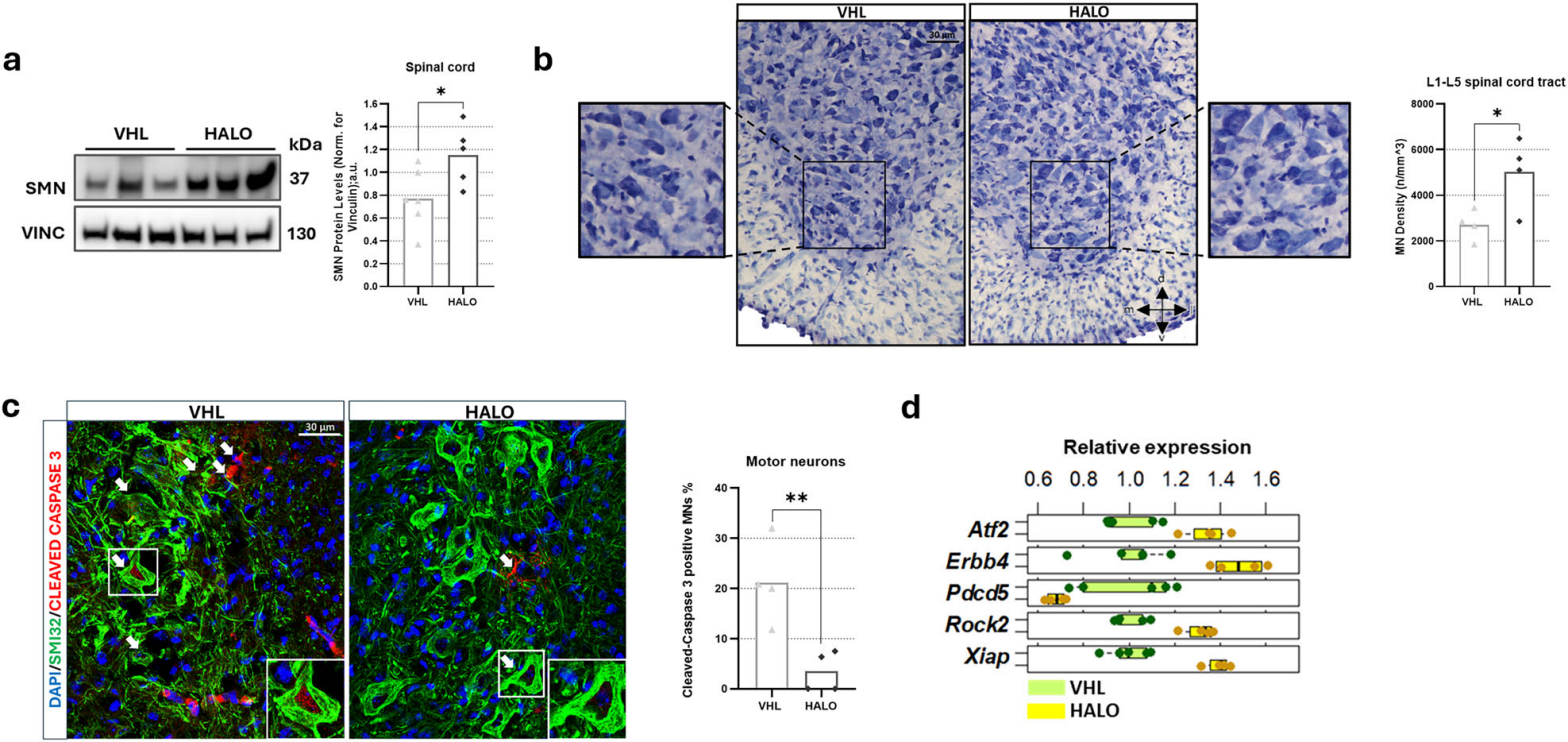
HALO improves SMN expression and MN survival in the spinal cord of delta 7 mice. **a** Representative densitometry (left) and quantification (right) of survival motor neuron (SMN) protein levels in vehicle (VHL)-treated and haloperidol (HALO)-treated delta 7 spinal cord. Vinculin (VINC) protein levels were referred as loading control; data are shown as mean from the individual animals, VHL *n* = 6, HALO *n* = 5, Student’s *t*-test, *P < 0.05. **b** Stereological lumbar alpha motor neuron (MN) counts in the spinal cord of VHL and HALO delta 7 mice. Representative images of Nissl-stained L1 spinal tract sections are shown. MN density (tract L1–L5) was assessed, *n* = 4 per group, Student’s *t*-test, *P < 0.05. **c** Representative confocal images showing MNs (SMI32-positive) and cleaved caspase 3-positive cells in ventral horns. The percentage of cleaved caspase 3-positive MNs was calculated; data are shown as mean from the individual animals, *n* = 4 per group; Student’s *t*-test, *P < 0.05, **P < 0.01. **d** Genes involved in apoptosis significantly regulated by HALO were identified in spinal cord RNA-sequencing data, according to GO function lists.

We previously showed that HALO increases SMN protein levels in patient fibroblasts in vitro^12^. Western blot analysis of the spinal cord and motor cortex in VHL-treated and HALO-treated delta 7 mice with SMA (VHL *n* = 6, HALO *n* = 5) confirmed these effects in vivo, with SMN protein levels rising by ~50% in the spinal cord (Fig. 2a) and ~25% in the motor cortex (Supplementary Fig. 2) compared with controls. A trend toward increased SMN levels was also observed in the liver and heart of HALO-treated delta 7 mice with SMA, although this did not reach statistical significance (Supplementary Fig. 3).

Given the observed motor improvements in delta 7 mice and the SMN expression increase in the central nervous system, we then investigated the neuroprotective effects of the drug treatment by evaluating MN survival. With this aim, stereological MN counts and quantification of apoptotic profiles in the HALO-treated and VHL-treated mice were performed, analyzing the lumbar (L1–L5) spinal cord tract (mice *n* = 4 per group). HALO-treated mice exhibited a significantly higher MN density (5,012 ± 773.4 (SEM) MNs/mm^3^) compared with VHL (2697 ± 330 (SEM) MNs/mm^3^) (Fig. 2b), confirming the treatment efficacy in delaying the SMA-associated cell death of MNs. Further supporting these observations, co-staining analysis for cleaved caspase 3 (apoptotic marker)^24^ and SMI32 (a marker of non-phosphorylated neurofilaments, enriched in α-MNs and facilitating their identification based on staining, cell size, and location) in the lumbar tract of the spinal cord ventral horns (Fig. 2c) revealed a significant reduction in apoptotic-positive MNs in HALO mice compared with controls (HALO: 3.46 ± 2.01%; VHL: 21.15 ± 4.15%, mice *n* = 4 per group).

In addition, RNA-seq analysis of the spinal cord revealed hundreds of differentially expressed genes in response to HALO (VHL *n* = 5; HALO *n* = 4; Supplementary Fig. 4a,c; see Supplementary Table 2 for full names) identifying upregulated neuroprotective pathways (*Erbb4, Atf2,* and *Rhock2*)^25–28^ and apoptotic regulators (*Xiap* upregulation and *Pdcd5* downregulation)^29,30^ (Fig. 2d), suggesting that HALO enhances SMN levels while activating neuroprotective and synaptic remodeling pathways to delay MN degeneration.

**Fig. 3:**
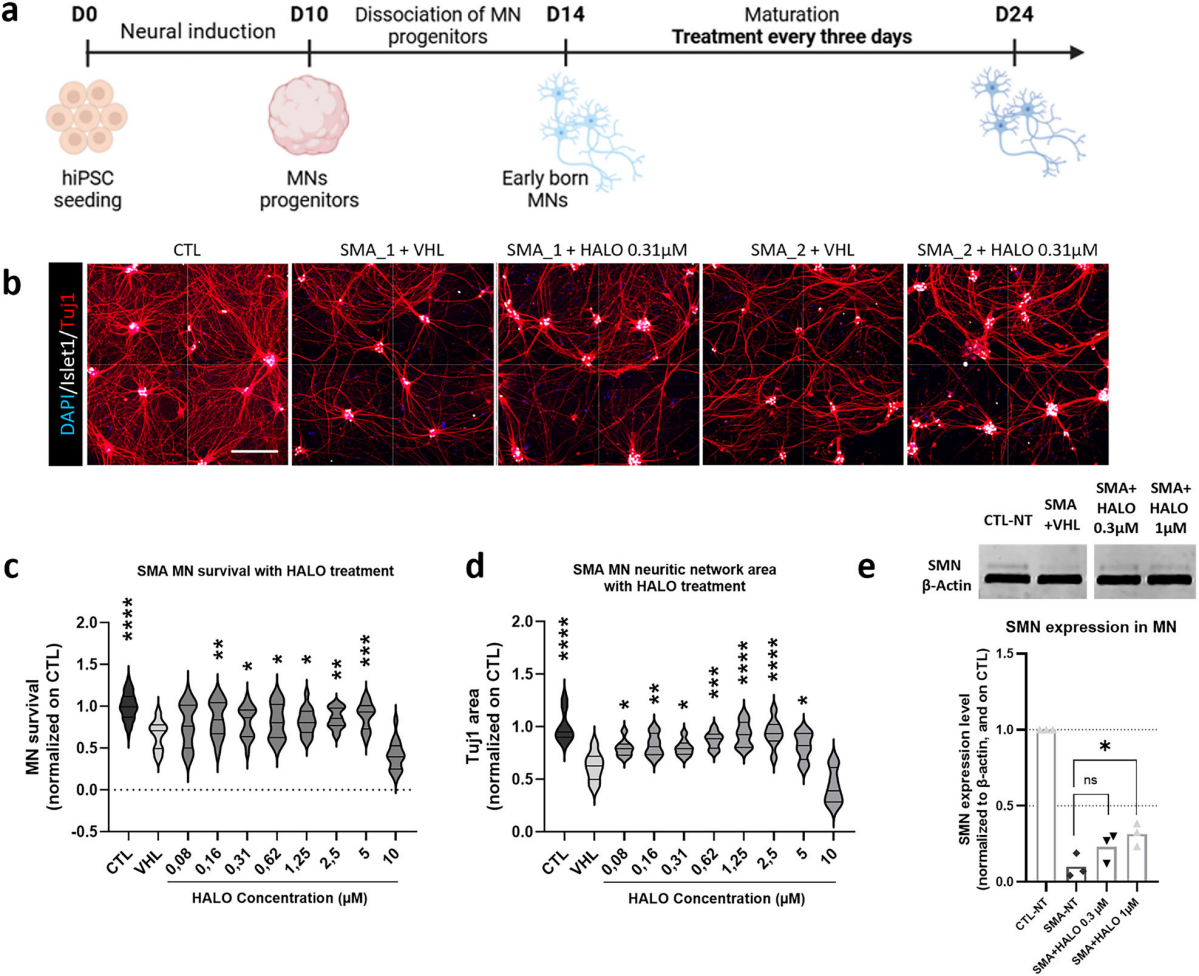
HALO increases SMN expression and improves MN survival in SMA patient-derived iPSCs. **a** Schematic view of the protocol (adapted from^30^) used to characterize spinal muscular atrophy (SMA) motor neuron (MN) survival (image created with BioRender.com). **b** Immunostaining of MNs at day 24. From left to right: CTL (control) MNs; cell line 1 SMA MNs receiving vehicle (VHL) or treated with haloperidol (HALO) at 0.31 µM; cell line 2 SMA MNs receiving VHL or treated with HALO at 0.31 µM. Scale bar, 100 µm. **c** Quantification of MN survival after treatment with different doses of HALO. Data are represented as violin plots of four independent experiments with four technical replicates each. **d** Quantification of MN neuritic network area (Tuj1-positive) after treatment with different doses of HALO. Data are represented as violin plots of two independent experiments with four technical replicates each. **e** Quantification of survival motor neuron (SMN) proteins by western blot. Data are displayed as the average SMN proteins, normalized to β-actin and on CTL not treated (NT) cells, measured by three independent experiments, indicated by dots. All statistics were calculated using one-way analysis of variance Dunnett’s multiple comparisons tests. ns, not significant. *P > 0.05, **P < 0.01, ***P < 0.001, ****P < 0.0001. DAPI, 4′,6-diamidino-2-phenylindole; hiPSC, human induced pluripotent stem cell.

To determine whether these findings translate to human in vitro systems, we tested HALO in hiPSC-derived MNs from patients with SMA (Fig. 3). Spinal MN differentiation was performed as described in^31^ (Fig. 3a). SMA hiPSC-derived MNs (Fig. 3b) exhibit typical disease-associated cell death, without exposure to stress factors, and a drastically reduced expression of the SMN protein compared with healthy control cells^13^ (Fig. 3c). To evaluate whether HALO can rescue this mortality, hiPSC-derived MNs were treated every 3 days during the 10 days of maturation phase, with different doses from 0.08 µM to 10 µM of HALO (Fig. 3c). Mortality was evaluated at the end of the maturation phase in comparison to the early-born MN viability by quantification of ISL+ cells (Supplementary Fig. 5). HALO prevented the hiPSC-derived MN mortality at doses ranging from 0.16 µM to 5 µM (Fig. 3d). A comparable effect occurred with risdiplam treatment from 0.125 µM to 0.25 µM (Supplementary Fig. 6a,b) and nusinersen at 0.25 µM (Supplementary Fig. 6a,c). Further experiments were performed at two doses found to improve the viability and networking of cells, namely, 0.31 µM and 1 µM. SMN expression analysis in treated and untreated hiPSC-derived MNs revealed that 1 µM of HALO treatment significantly increased SMN levels, although it did not restore it up to the healthy control level (Fig. 3e). In comparison, a similar in vitro treatment of hiPSC-derived MNs with 0.13 µM risdiplam or 0.25 µM nusinersen did not significantly increase SMN protein at the end of the maturation phase (Supplementary Fig. 6d).

### HALO modulates alternative splicing programs of direct SMN targets in the spinal cord of mice with SMA

**Fig. 4:**
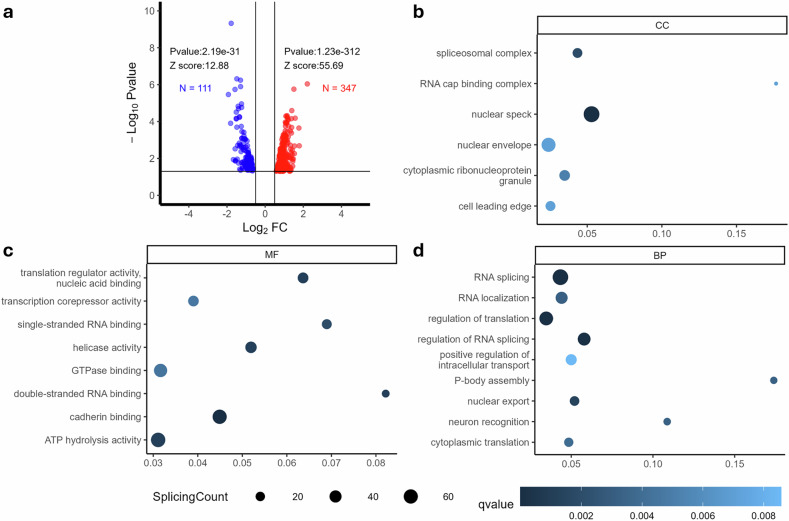
Analysis of alternative splicing changes in SMN direct target genes in response to HALO treatment. **a** A total of 458 splicing events exhibit significant changes of >40%. These events can be under-represented (blue) or over-represented (red). Each splicing event is depicted with its individual *P*-value (−log_10_ *P*-value, *y*-axis) and fold change (log_2_FC, *x*-axis). These splicing alterations correspond to 139 genes, which are classified based on their functional annotation (parts **b–d**). The dot plots represent the number of splicing events per category in cellular component (CC) (part **b**), molecular function (MF) (part **c**), and biological process (BP) (part **d**). The dot size corresponds to the number of events, whereas the color indicates the level of significance based on the *q*-value. HALO, haloperidol; SMN, survival motor neuron.

To further investigate the molecular mechanisms underlying neuroprotective effects of HALO, we performed RNA-seq analysis on spinal cords from mice treated for SMA focussing on the impact of HALO on alternative splicing regulation (VHL *n* = 5; HALO *n* = 4). A volcano plot of differentially spliced events (Fig. 4a) highlights significant enrichment by HALO treatment of direct SMN target transcripts. Among the 1,336 known SMN target genes, 139 exhibited significant splicing changes (≥40% change), in response to treatment, with a total of 458 events of splicing (Supplementary Table 3). RNA‑seq analysis did not reveal significant changes in overall SMN expression nor any increase in *SMN2* exon 7 inclusion (Supplementary Fig. 7). By contrast, we observed a borderline significant increase in exon 8 inclusion (1,407-fold change; *P* = 0.058). Recent studies^32^ suggest that exon 8 splicing can enhance SMN mRNA stability and protein half‑life, providing a plausible explanation for improved protein levels even in the absence of detectable changes in exon 7 inclusion. Functional annotation of HALO-dependent splicing events revealed significant clustering in key molecular functions, cellular components, and biological processes, with strong effects on RNA metabolism and intracellular transport (Fig. 4b–d). Notably, single-stranded RNA binding and translation regulator activity were altered, suggesting broader post-transcriptional disruptions in SMA that HALO may mitigate. Splicing changes were concentrated in nuclear specks and the spliceosomal complex, both essential for RNA processing, and the disruption of spliceosomal genes further supports the role of HALO in restoring small nuclear ribonucleoprotein homeostasis and correcting alternative splicing defects^33^. RNA splicing, its regulation, and localization were among the most affected processes — closely linked to SMN function, whose deficiency disrupts small nuclear ribonucleoprotein biogenesis and splicing fidelity. Restoration of these programs suggests that HALO may counteract a core molecular defect in SMA, supporting proper transcript processing for neuronal survival.

### HALO treatment modulates neuroinflammatory responses in the murine spinal cord

**Fig. 5:**
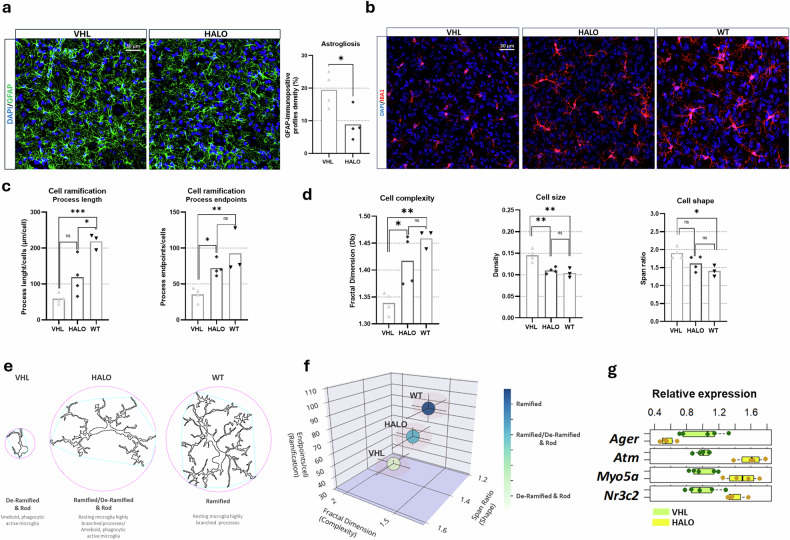
Effect of HALO on neuroinflammation in the ventral horns of delta 7 mice lumbar spinal cord. **a** Representative confocal image of glial fibrillary acidic protein (GFAP)-positive astrocytes in the ventral horns (left) and quantification of GFAP-immunopositive profile density in haloperidol (HALO) mice versus vehicle (VHL) group (right). Data are presented as mean ± SEM per individual animal, *n* = 4 per group, Student’s *t*-test, *P < 0.05. **b** Representative confocal images of IBA1-positive microglial cells in HALO-treated/VHL-treated and wild-type (WT) mice samples. **c** Summary data of microglia cell ramification, data are expressed as mean cell process length/endpoint per individual animal. **d** Summary data of microglia cell complexity/size/shape. Data are expressed as mean cell fractal dimension/density/span ratio per individual animal. **c**,**d**, One-way analysis of variance, followed by Tukey’s multiple comparison post hoc test; VHL/HALO *n* = 4, WT *n* = 3; *P < 0.05, **P < 0.01, ***P < 0.005, ***P < 0.0001. **e** Schematic cell fractal dimension reconstructions of microglial cell phenotypes. **f** 3D-scatterplot showing the skeleton and the fractal analysis data relationship. **g** Spinal cord RNA-sequencing data for neuroinflammation-related genes significantly modulated by HALO treatment. The gene list was manually curated using GO terms and the Molecular Signature Database, adjusted *P*-value < 0.05. DAPI, 4′,6-diamidino-2-phenylindole.

Together with MN degeneration, SMA is characterized by increased neuroinflammation. Several studies in SMA in vivo and in vitro models highlighted the possible contribution of active astrocytes^7,8^ and phagocytic microglia cells as SMA neuropathology modifiers, and both astrogliosis and reactive morphology microglia were found prominently in end-stage delta 7 mice spinal cords^7–9,34^. To evaluate whether increased SMN levels modulate neuroinflammation, we assessed astrogliosis and microglial morphology (GFAP and IBA1 immunostainings, respectively) in the lumbar spinal cord ventral horns using confocal microscopy. HALO treatment significantly reduced GFAP-immunopositive profile density by ~37% in delta 7 mice compared with controls (mice *n* = 4 per group) (Fig. 5a), suggesting that astrocyte activation modulation may contribute to — or result from — HALO-dependent MN preservation.

Subsequently, we performed an in-depth morphological characterization and classification of microglial cell phenotypes in HALO-treated and VHL-treated mice samples. To clearly discriminate different degrees of cellular active state compared with that of resting ones (WT condition), we performed a comparative morphological analysis of the microglia in treated HALO and VHL mice and WT controls (VHL *n* = 4, HALO *n* = 4, WT *n* = 3) (Fig. 5b). First, we assessed that there was no difference in the number of IBA1-positive microglial cells in HALO-treated mice compared with VHL mice (Supplementary Fig. 8). Microglial morphology was analyzed using skeletal and fractal analyses to assess branching, complexity, size, and shape across VHL-treated, HALO-treated, and WT mice. Delta 7 mice treated with VHL exhibited significant differences in microglial branching, complexity, size, and shape compared with WT controls. Detailed characterization confirmed a more reactive morphology in SMA-VHL microglia (amoeboid, de-ramified, and rod-like, associated with the active phagocytic state) versus the highly branched, thin-soma morphology of WT microglia, indicative of the resting state^35^ (Fig. 5c–f). Interestingly, HALO mice showed a significant improvement in branching (more than twofold increase in terms of process end points) and overall microglial complexity compared with VHL mice, while also showing nonsignificant differences in process length, cellular complexity, size, and shape compared with WT controls (Fig. 5c,d). Data on cell branching, cell complexity, and shape were further averaged in each group for Pearson's correlation^35^ to obtain a different classification of microglial morphology in mice treated for SMA compared with WT controls. HALO mice microglial cells revealed a “ramified/de-ramified & rod” phenotype, significantly less reactive than VHL “de-ramified & rod” microglia and close to the morphology of WT control resting microglia (Fig. 5e,f).

RNA-seq analysis further confirmed the significant impact of HALO on some key neuroinflammation-related genes (VHL *n* = 5; HALO *n* = 4; Fig. 5g; see Supplementary Table 2 for full names). Notably, *Ager*, a key promoter of neuroinflammation^36^, was downregulated in HALO-treated spinal cords. Conversely, *Atm*, *Myo5a*, and *Nr3c2* —associated with neuroprotection, DNA repair, and anti-inflammatory signaling — were all upregulated.

Overall, these findings support that HALO has a modulatory effect on the neuroinflammatory pathways of astrogliosis and reactive microglia, possibly helping to mitigate the latter’s impact on SMA neuropathology.

### HALO improves SMN expression, muscle trophism, and NMJ phenotype in mice with SMA and in patient‑derived cells

**Fig. 6:**
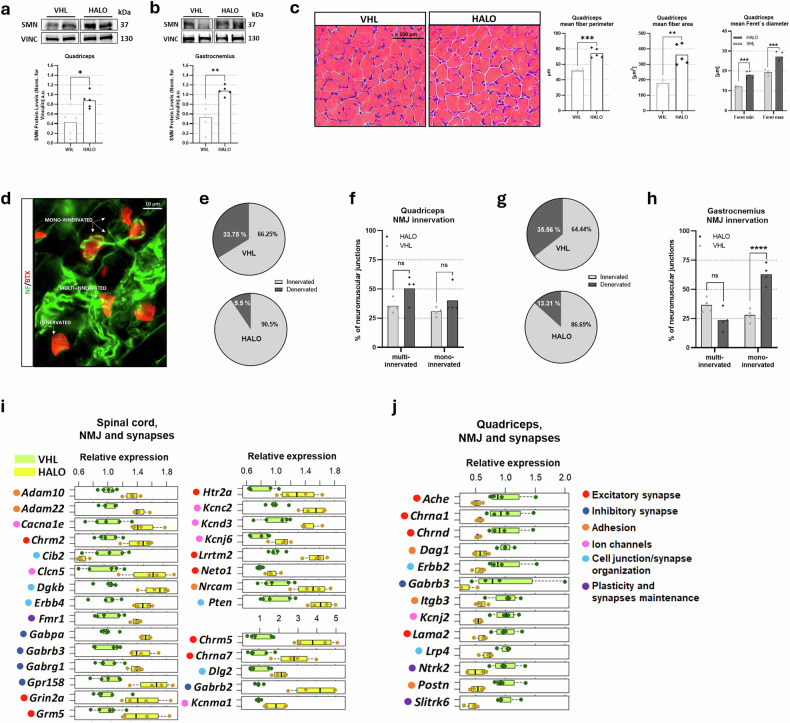
HALO treatment improves SMN expression, muscle fiber morphology, and NMJ phenotypes in mice with SMA. Representative immunoblots of SMN protein levels in vehicle (VHL)-treated and haloperidol (HALO)-treated quadriceps (part **a**) and gastrocnemius (part **b**). Vinculin (VINC) protein levels were referred as loading control; data are shown as mean per individual animal, *n* = 5 per group, Student’s *t*-test. **c** Representative hematoxylin and eosin-stained quadriceps sections of treated mice. The muscle fiber area, perimeter, and Feret’s diameter are reported. Student’s *t*-test. **d** Representative immunofluorescence images show double staining of endplates (BTX) and neurofilament (NF), allowing neuromuscular junction (NMJ) phenotype discrimination. **e–h** NMJ phenotype percentages are described for quadriceps/gastrocnemius in pie charts for innervated/denervated NMJs (parts **e** and **g**) and in plots for monoinnervated/multi-innervated NMJs (parts **f** and **h**); two-way analysis of variance, followed by Sidak’s multiple comparison post hoc test. For parts **c** and **e–h** data are shown as the averages obtained per individual animal, *n* = 4 per group. For parts **a–c**, **f**, and **h**, *P < 0.05, **P < 0.01, ***P < 0.005, ****P < 0.001. Spinal cord (part **i**) and quadriceps (part **j**) RNA-sequencing data for NMJ constitution and synaptic function-related genes significantly modulated by HALO treatment. All genes in parts **i** and **j** were normalized to VHL value average, according to adjusted *P*-value <0.05. ns, not significant.

Given the positive effects on motor behavior and SMA spinal cord pathology, we next assessed HALO’s impact on skeletal muscle by analyzing SMN expression, muscle trophism, and NMJ innervation in the quadriceps and gastrocnemius. Immunoblotting revealed a significant SMN increase (≥100%) in HALO-treated skeletal muscles (mice *n* = 5 per group) (Fig. 6a,b), mirroring the spinal cord findings and confirming that HALO enhances SMN levels peripherally.

Prompted by these positive results, we then verified the efficacy of HALO on muscle trophism. To this aim, we first performed morphological analyses of the hematoxylin and eosin-stained quadriceps and gastrocnemius muscles at P12 (mice *n* = 4 per group) (Fig. 6c). We evaluated the mean fiber area, perimeter, and the Feret’s diameters of 100 muscle fibers per animal (Supplementary Tables 4 and 5). In HALO-treated quadriceps, the results showed a significant increase in fiber area (+104%), fiber perimeter (+43%), and Feret’s diameters (min Feret: +48% and max Feret: +40%). Regarding the analysis of gastrocnemius morphology, we did not observe differences in fiber parameters in HALO-treated mice compared with VHL animals.

RNA-seq analysis of quadriceps revealed differentially expressed genes following HALO treatment (VHL, *n* = 4; HALO *n* = 4) (Supplementary Fig. 4b). The analysis highlighted an impact on adipogenesis (Supplementary Fig. 4d). To explore this, we examined lipid metabolism and adipogenesis-related genes, identifying 11 genes related to lipid anabolism, all significantly downregulated (Supplementary Fig. 9). This suggests HALO treatment may promote lipid and adipocyte depletion as part of the muscle’s adaptive response.

NMJ innervation and morphology were assessed in quadriceps and gastrocnemius muscles from P12 delta 7 mice treated with HALO or VHL (mice *n* = 4 per group). NMJ innervation was analyzed for the number of neurofilaments contacting the endplate (Fig. 6d); in quadriceps, HALO treatment significantly reduced denervated NMJs (72%) and increased innervated junctions (+36%) (Fig. 6e) without altering monoinnervation or multi-innervation levels (Fig. 6f). In gastrocnemius, HALO similarly decreased denervated NMJs (−63%) and increased innervated NMJs (+34%) (Fig. 6g), while also reducing multi-innervated NMJs (HALO: 27.35 ± 5.23%; VHL: 56.50 ± 4.01%) and increasing monoinnervated NMJs (HALO: 72.65 ± 5.23%; VHL: 43.50 ± 4.01%) in comparison with the VHL group, suggesting the positive effect of HALO on NMJ maturation in this tissue (Fig. 6h). Furthermore, RNA-seq analysis revealed differential regulation of genes associated with NMJ formation and function, including those involved in synaptic transmission, ion channel activity, and cell adhesion. These genes were upregulated in the spinal cord (Fig. 6i and Supplementary Table 2) but downregulated in quadriceps (Fig. 6j and Supplementary Table 6), suggesting tissue-specific NMJ remodeling in response to treatment (spinal cord: VHL *n* = 5, HALO *n* = 4; quadriceps: HALO *n* = 4, VHL *n* = 4). This divergence may result from HALO’s selective action through DRD2, which are highly expressed in the central nervous system, but minimally present in skeletal muscle^37^. In fact, our RNA-seq data confirmed expression of dopamine receptors DRD1 (ENSMUSG00000021478), DRD2 (ENSMUSG00000032259), and DRD5 (ENSMUSG00000039358) in spinal cord samples (Supplementary Data File 1), whereas these receptors were completely undetectable in quadriceps. Additionally, given that synaptic signaling is a highly directional process, the observed tissue-specific transcriptional responses likely reflect fundamental differences in how MNs and peripheral muscle tissue adapt to pharmacological modulation.

Overall, HALO administration in delta 7 mice supports skeletal muscle trophism, promotes NMJ maturation, enhances innervation, and increases fiber size.

**Fig. 7:**
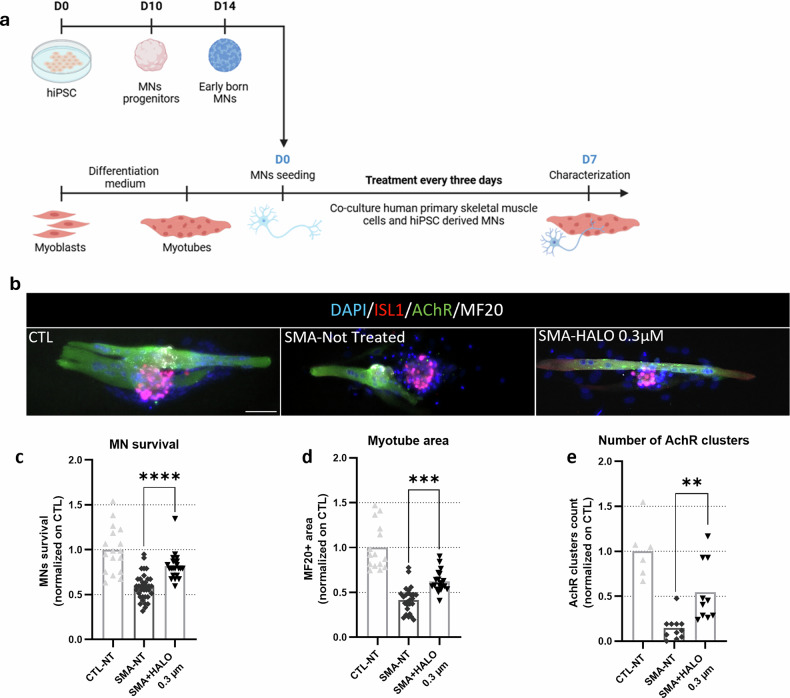
HALO promotes MN survival, myotube fusion, and AchR clustering in an SMA co-culture model. **a** Schematic view of the myotubes and motor neuron (MN) co-culture protocol (image created with BioRender.com). **b** Immunostaining of co-cultures at day 7, comparing control (CTL)-immortalized myotubes co-cultured with CTL human induced pluripotent stem cell (hiPSC)-derived MNs and spinal muscular atrophy (SMA)-immortalized myotubes co-cultured with SMA hiPSC-derived MNs, with or without haloperidol (HALO) treatment. Scale bar, 100 µm. Data in the indicated experimental groups are displayed for quantification of MN survival in CTL and SMA co-cultures (part **c**); quantification of fusion of immortalized myotubes in co-culture CTL or SMA (part **d**); and quantification of acetylcholine receptor (AchR) cluster in CTL or SMA co-cultures (part **e**). Data are displayed as histograms of the averaged measures and single dots of each technical replicate. Statistical analysis was performed as an ordinary one-way analysis of variance, Dunnett’s multiple comparisons test. *P < 0.05, **P < 0.01, ***P < 0.001, ****P < 0.0001. DAPI, 4′,6-diamidino-2-phenylindole; ns, not significant; NT, not treated.

In vitro, the SMA-related muscle atrophy phenotype is translated into a significant reduction in the size of SMA myotubes. To assess effects of HALO on SMA myotubes and NMJs, we used a co-culture system of hiPSC-derived MNs and micropatterned immortalized myotubes, providing a robust and reproducible in vitro NMJ model. Immortalized myoblasts were differentiated into single myotubes on micropatterned dishes, whereas hiPSCs were differentiated into early-born MNs and seeded onto them (Fig. 7a). Healthy control co-cultures were obtained by using control hiPSC and immortalized myoblasts, whereas SMA co-cultures were obtained by using hiPSC and immortalized myoblasts derived from patients with SMA (Fig. 7b). These co-cultures were used to evaluate the effects of HALO in an NMJ context, on MN survival, myotubes size, and the area covered by acetylcholine receptors (AchR) clusters (Fig. 7c–e). Since HALO’s important side effects are well known, the effect of a low dose is particularly of interest. Hence, cell co-cultures were exposed to a low HALO dose of 0.3 µM, already improving hiPSC-derived MNs survival and networking. Notably, untreated SMA co-cultures exhibited the typical reduced myotubes size, the increased mortality of MNs, and a reduced number of AchR clusters. The HALO treatment at 0.3 µM significantly rescued all of these phenotypes, confirming its beneficial effect on SMA cells and particularly in the neuromuscular interaction system.

### Haloperidol effects on mitochondrial gene expression and the dopamine receptor

HALO has been shown to induce changes in mitochondrial structure and function, including time-dependent and dose-dependent inhibition of complex I (NADH ubiquinone oxidoreductase) in murine models^38^. Mitochondrial dysfunction is a well-known hallmark of SMA, with evidence of decreased membrane potential (ΔΨm) and respiration^39,40^, likely due to altered regulation of electron transport chain (ETC) complexes — particularly complex I — and ATP production^39,40^ in both murine and human stem cell-derived SMA MNs.

**Fig. 8:**
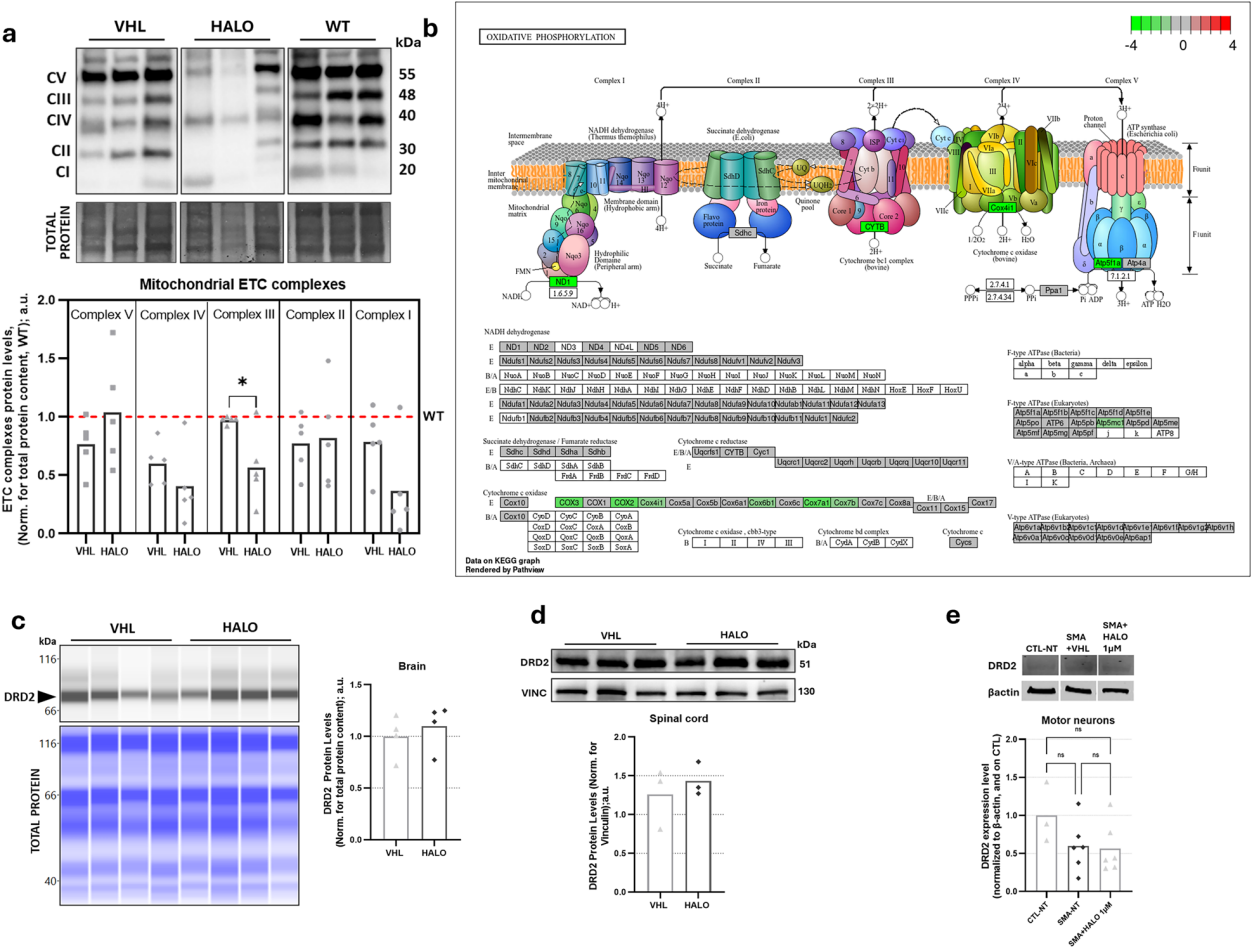
Investigations of HALO impact on mitochondrial ETC complexes and DRD2 expression in treated SMA samples. **a** Haloperidol (HALO) effects on mitochondrial electron transport chain (ETC) complexes in murine spinal cord. Immunoblotting analysis of mitochondrial ETC complex protein levels in vehicle (VHL)-treated and HALO-treated spinal muscular atrophy (SMA) spinal cords, compared with wild-type (WT). Representative densitometries are shown (up); total protein staining (Coomassie) was referred as loading control. Data are shown as mean from the individual animals, normalized to the WT sample mean (red dashed lines), VHL *n* = 5, HALO *n* = 5, WT *n* = 3; Student’s *t*-test. **b** In spinal cord RNA-sequencing data, gene enrichment analysis was conducted in DAVID (https://david.ncifcrf.gov) using KEGG pathway annotation, identifying downregulation of mitochondrial ETC components. DRD2 protein levels in delta 7 brains (part **c**, Jess analysis; VHL *n* = 4, HALO *n* = 4) and spinal cord (part **d**, western blot analysis; VHL *n* = 3, HALO *n* = 3), and in patient-derived MNs (part **e**, western blot analysis; SMA NT *n* = 3, SMA + HALO 0.3 µM, SMA + HALO 1 µM, *n* = 6). Representative densitometries are shown on the right. Total protein staining was referred as loading control. Data are shown as mean from the individual samples. CTL, control; DRD2, dopamine D2 receptor; ns, not significant; VINC, vinculin.

To evaluate the impact of HALO on SMA mitochondrial dysfunctions, we investigated mitochondrial ETC complex protein levels in spinal cord samples of treated mice (VHL *n* = 5, HALO *n* = 5, WT *n* = 3). We observed significant differences between HALO-treated mice and VHL ones in the complex III protein levels (−42%) (Fig. 8a); transcriptomic analysis confirmed these findings, indicating a downregulation of genes encoding ETC complexes, particularly those related to complex III (VHL *n* = 5, HALO *n* = 4) (Fig. 8b). Our findings suggest changes related to mitochondrial function in response to treatment but we cannot define in which direction these changes occur, as many other mitochondrial defects have been described in SMA neural cells. It is well established that MNs in patients with SMA exhibit pronounced mitochondrial fragmentation and defects in mitochondrial fusion and location^39,40^. Different alterations in mitochondrial dynamics beyond bioenergetic deficits may also be involved in HALO’s effects on the SMA spinal cord, which cannot be elucidated from our current study. This represents an interesting direction for future research.

HALO can modulate DRD2 expression in rats in an apparent dose-dependent manner, increasing *DRD2* mRNA at 2 mg/kg in the anterior cingulate cortex^41^ while reducing it at 10 mg/kg in the striatum^15^, underscoring the need for further assessment under our experimental conditions. To investigate whether the lower concentration of HALO used in this study (0.5 mg/kg/day) could similarly impact DRD2 expression in SMA models, we assessed DRD2 protein levels in treated mice brain and spinal cord samples and in hiPSCs-derived MNs. HALO treatment did not significantly affect DRD2 protein levels in the analyzed samples (Fig. 8c–e), consistent with RNA-seq data (Supplementary Fig. 10), suggesting that at therapeutically effective concentrations the action of HALO seems to be independent of DRD2 expression changes. Consistently, the neuromuscular benefits of HALO in mice with SMA appear largely disease-dependent rather than reflecting nonspecific DRD2-mediated effects. To further support this conclusion, we provide additional data from WT mice treated with HALO (0.25 mg/kg) (Supplementary Fig. 11a–c). Importantly, HALO did not significantly affect body weight (Supplementary Fig. 11b) or SMN protein levels in WT mice (Supplementary Fig. 11c), supporting limited impact under physiological conditions and reinforcing the interpretation that HALO primarily corrects disease-specific deficits in SMA.

## Discussion

Here, we report further investigations on the effect of daily HALO administration in a severe SMA mouse model and in patient-derived MNs and myotubes, demonstrating the efficacy of the treatment in rescuing SMA phenotypes in both models. HALO is recognized for its therapeutic effect through dopamine receptor antagonism, particularly DRD2, which remains a well-established mechanism^14^. However, HALO also binds with high affinity to sigma-1 receptors (S1Rs), a chaperone implicated in neuroprotection and cellular stress responses^42^. This interaction may influence tissue-specific activity. Moreover, dopamine receptor signaling itself depends on tissue context and receptor oligomerization, and HALO may act on DRD1/DRD2 heterodimers that interact with S1R, modulating phosphorylation cascades such as adenylyl cyclase and MAPK. RNA-seq of spinal cords of HALO-treated mice with SMA showed extensive splicing changes, including SMN target transcripts, indicating enhanced SMN activity. Functional enrichment highlighted genes involved in splicing, transcription and translation, and energy metabolism, suggesting broader neuronal effects. Notably, despite this broad splicing signature, our RNA-seq data did not show increased SMN expression or *SMN2* exon 7 inclusion in Δ7 mice, but instead pointed to changes consistent with alternative mechanisms (for example, a trend toward higher exon 8 inclusion), which has been linked to enhanced SMN mRNA stability and protein half-life and could explain improved protein levels without detectable exon 7 correction^32^. Consistently, high-dose HALO in human SMA fibroblasts increased both full-length-SMN and Δ7 SMN isoforms, supporting a model in which HALO promotes SMN rescue via broader post-transcriptional effects on transcript abundance/stability rather than canonical exon 7 splicing correction^12^.

These findings provide strong molecular evidence for role of HALO in modulating SMN-dependent pathways, highlighting its potential as a therapeutic for SMA. They also reveal the impact of HALO on multiple pathways, improving disease-related pathological signs in MNs and skeletal muscles, offering new insights into ongoing exploration of their role in SMA neurodegeneration.

### HALO exerts neuroprotective effect in spinal cord of mice with SMA and in patient-derived cells

HALO treatment prevented mice early death events and achieved a mean lifespan of 15 days, a remarkable result, based on the extremely short lifespan of delta 7 mice. Subsequently, we also confirmed in the murine model the efficacy of HALO in significantly increasing SMN expression in different tissues (spinal cord, quadriceps, and gastrocnemius). This SMN-enhancing effect in mice treated for SMA resulted in significantly improved behavioral outcomes and motor performance, delay in neurodegeneration, and enhanced muscle trophism and NMJ phenotypes.

In detail, in spinal cord ventral horns, we assessed the SMN-dependent effect of HALO in delaying lower MN degeneration versus VHL, both by stereological analysis showing higher MN density in the entire lumbar tract and by confirming decreased MN positivity to pro-apoptotic markers (cleaved caspase 3). SMN deficiency strongly correlates with gene dysregulation triggering programmed cell death, causing persistent abnormal MN degeneration (for example, *BAX*, *BCL-XL*, *BCL2*, *NAIP*, and *XIAP*)^2,21,24,43,44^ and increased caspase-3 cleavage^21,24^. Our results further suggest that HALO exerts an SMN-dependent effect on genes involved in regulating apoptosis, promoting the overexpression of the anti-apoptotic *Xiap* and the downregulation of the pro-apoptotic *Pdcd5* (Supplementary Table 2). The latter, among several cell death-related proteins, regulates the activity of caspase 3/7, BCL-2, and BAX^29,30^, further strengthening our observations on the treatment efficacy in reducing the apoptosis executioner cleaved caspase 3 in spinal MNs. Moreover, the SMN-dependent modulation elicited by HALO may also help stabilize defective spinal cord mitochondrial pathways implicated in triggering intrinsic apoptosis, as shown in SMA models and patients, where altered mitochondrial pro-apoptotic proteins such as BCL2, P53, and cytochrome *c*, together with mitochondrial dysfunction-dependent caspase activation, contribute to disease progression^40^. HALO also upregulates genes supporting MN maturation and function (*Erbb4*, *Atf2*, and *Rhock2*), a key target of ongoing therapeutic research in SMA^25,26^, suggesting that its effects also extend to other pathways aiding neuroprotection. As example, as HALO treatment was reported into enhancing NRG1/ErbB signaling^45^, the HALO-induced *Erbb4* upregulation in the spinal cord could contribute to SMA motor axon maturation and maintenance, aligning with previous studies on NRG1/ErbB signaling in MN activity^25,26^.

HALO was able to restore the survival also in iPSC-derived MNs from patients with SMA and their ability to form neuritic networks, reaching levels comparable to healthy cells. Similar results were seen with a treatment of two drugs for SMA: risdiplam and nusinersen. Notably, SMN protein was restored only to ~32% of healthy levels. Both risdiplam and nusinersen also caused a mild SMN increase during maturation phase, suggesting that even a small SMN rise can significantly improve that SMA iPCS-derived MN survival, as previously observed in a study exploring the potential of moxifloxacin treatment for SMA^13^.

### HALO treatment reduces neuroinflammation and preserves MN survival

It is noteworthy that neuroprotective effects of HALO in delaying MN degeneration could also be linked to the modulation of the neuroinflammatory response in the spinal cord of treated mice. Furthermore, cell death and neuroinflammation processes are strongly interconnected^46^, and it has been shown that SMN deficiency is also upstream of glial cell (astrocytes and microglia) dysfunctions, known to exacerbate the SMA condition^7–9,34^. Our analysis showed a significant decrease in astrogliosis in HALO mice ventral horns compared with controls and provided new insights into the microglia activation in mice with SMA. We correlated the morphological clustering of microglia cells in WT and mice untreated for SMA with the cell-active state degree (as performed for other neuronal disease models^35^), highlighting that HALO mice show a less-reactive microglia phenotype in comparison to VHL and no significant differences with WT ones. The effects of HALO on neuroinflammation could be attributed to its ability to attenuate pro-inflammatory processes in glial cells. Indeed, HALO (and its metabolites) was shown to boost S1R-mediated brain-derived neurotrophic factor release, decrease abnormal S100B levels in astrocytes, and reduce inducible nitric oxide synthase and tumor necrosis factor-α expression, thus preventing interferon-γ-induced microglial activation^47,48^. Moreover, because S1R is predominantly located at mitochondria-associated ER membranes, where it regulates Ca^2+^ transfer, mitigates mitochondrial stress, and modulates intrinsic apoptotic signaling^49^, all pathways known to be impaired in SMA spinal cord MNs^40^, its engagement by HALO may further contribute to restoring mitochondrial homeostasis and limiting apoptosis-related inflammatory responses.

Consistently, we observed that HALO modulates neuroinflammation regulatory genes in treated mice. HALO downregulates the *Ager* gene, which encodes a protein involved in activating NF-kappaB and releasing pro-inflammatory cytokines (IL6, IL8, and tumor necrosis factor-α)^36^, contributing to microglia and astrocyte reactivity. Interestingly, we observed that HALO also induces the upregulation of genes that, while acting on different cellular pathways, have all been identified as targets for reducing glial-driven neuroinflammation in neurodegenerative diseases. As an example, *ATM* deficiency is known to activate microglia, causing neuronal damage and neurodegeneration, and also to impair growth and induce oxidative stress, endoplasmic reticulum stress, and ERK activation in astrocytes^50,51^.

### HALO attenuates muscle atrophy and improves NMJ phenotypes in SMA models

Concerning HALO treatment effect in skeletal muscles, we observed that SMN levels doubled, both in quadriceps and in gastrocnemius, with improved muscle fiber size and NMJ integrity. The effect of drug on muscle morphology was significant only in the quadriceps, a proximal muscle. As MNs innervating proximal muscles are selectively vulnerable in SMA neurodegeneration, we justify this selective trophism improvement as more evident in quadriceps that is earlier and more severely affected in the progression of the disease^13,52–55^. Moreover, in the quadriceps, HALO downregulates genes related to adipogenesis and fatty acid metabolism pathways, which are altered in SMN deficiency. As HALO has been shown to affect lipid homeostasis in multiple models^56,57^, we hypothesize that it may ameliorate the SMA dyslipidemia phenotype in skeletal muscles^58^, either directly or by increasing SMN protein levels, contributing to improved muscle trophism. Consistent with this, SMA skeletal muscle displays profound mitochondrial abnormalities, including impaired respiratory chain activity and altered FAO, which may contribute to dyslipidemia and could also be modulated by SMN-dependent effects of HALO on mitochondrial function^40^.

The NMJ analyses highlighted remarkable improvements both in the quadriceps and in the gastrocnemius, showing a significantly reduced percentage of denervated NMJs compared with the VHL group. Both muscles also showed improved motor endplate innervation/maturation, characterized by a higher percentage of monoinnervated NMJs, which occurred significantly in the gastrocnemius. Notably, the HALO treatment led to a significant increase in the expression of genes associated with synaptic plasticity in the spinal cord, as well as those involved in inhibitory and excitatory synapse receptors, differentiation, maintenance, and structure. Intriguingly, a subset of genes involved in similar pathways was found to be downregulated in the quadriceps, suggesting differential mechanisms of the drug in these tissues. These different responses could be explained by the fact that haloperidol is documented to act through DRD2, which is abundant in the spinal cord but barely expressed in skeletal muscle^59^, as also shown in our RNA-seq data. The fact that effects of dopamine on muscle are not blocked by DRD2 antagonists (such as haloperidol) but are blocked by DRD1 antagonists (such as SCH-23390) supports this notion^60^. Furthermore, the differential mechanisms and gene responses to the drug in spinal and muscle tissue may also reflect tissue-dependent effects of HALO-induced SMN upregulation on mitochondrial morphology and activity. In SMA, MNs exhibit fragmented axonal mitochondria with reduced complex I-dependent respiration, ATP production, and mitochondrial Ca^2+^ uptake, whereas skeletal muscle shows a collapsed intermyofibrillar network with mitochondrial DNA depletion, reduced respiratory chain activity, and marked alterations in fatty acid and glucose metabolism (reviewed elsewhere^40^). MNs are particularly dependent on SMN for axonal RNA metabolism and mitochondrial trafficking/Ca²⁺ handling; even a moderate HALO-induced increase in SMN may improve mitochondrial bioenergetics and Ca^2+^ handling in the spinal cord, thereby unlocking prosynaptic transcriptional programs such as the plasticity-related, receptor-related and differentiation-related genes that we find upregulated. By contrast, SMA skeletal muscle starts from a more metabolically constrained and structurally compromised mitochondrial state, and SMN upregulation may act on a primarily “energy-saving/remodeling” substrate rather than a plastic one, contributing to the differential gene regulation that we observe between spinal cord and quadriceps after HALO treatment. Synaptic dysfunction, characterized by loss of synaptic input to MNs, slowed postsynaptic potentials, reduced neurotransmitter release, and structural changes at the NMJ, is an early and critical pathological event in SMA that worsens motor deficits^55,61^. SMN has a pivotal role in enhancing the reciprocal signaling between MN and muscles, and the protein-restoring MNs have been shown to partially improve NMJ health and motor function in mice with SMA^22,61–64^. Therefore, strategies focussed on synaptic repair and broader SMN restoration emerge as key therapeutic targets for SMA. Herein, the remarkable results obtained in the improving of NMJ maintenance/maturation and synaptic gene expressions are reflected in mice motor functions and reasonably are due to the HALO-induced increase in SMN expression in several tissues. Furthermore, the effects of HALO treatment were associated with homeostatic changes and improved synaptic plasticity in mice, further adding to its candidacy for the treatment of SMA.

These observations have been also confirmed on a human in vitro system, by treating co-culture of hiPSC-derived MNs with primary skeletal cells: HALO improved the myogenic fusion of skeletal muscle cells and increased the number of AchRs, translating a better communication between both compartments.

### Limitations of the study

Although this study demonstrates the potential therapeutic benefits of HALO in treating SMA, some limitations must be acknowledged. First, the precise molecular mechanisms underlying the effects of HALO — beyond SMN upregulation — remain incompletely understood, particularly in relation to neuroprotection, NMJ remodeling, neuroinflammation, and metabolic and enhanced muscle morphology effects in skeletal muscle. Further studies should dissect these pathways and clarify whether HALO exerts direct or secondary effects on these processes. In addition, although HALO elicits a broad therapeutic response, its current dosing and delivery paradigm does not yet achieve the broad and tissue-wide restoration of SMN levels required for maximal lifespan extension in delta 7 mice, as that reported for SMN-restoring gene-based therapies such as nusinersen or risdiplam^65,66^.

Moreover, this study focuses on short-term efficacy in preclinical models, and long-term disease-modifying potential remains unknown. Future research should assess whether benefits persist, or compensatory mechanisms reduce efficacy. Furthermore, the RNA-seq data provide insights into gene expression changes but functional validation is needed to fully define the drug’s impact on SMA pathology. Finally, although HALO demonstrated no major side effects, in our models, its interaction with existing SMA therapies (for example, nusinersen, risdiplam, or gene therapy) requires further evaluation to exclude possible antagonistic or pharmacokinetic effects. Clinical trials will ultimately be necessary to determine HALO’s long-term safety and efficacy in patients with SMA.

### Therapeutic implications of the study

Our findings reinforce HALO’s safety and therapeutic potential, demonstrating no major drug-related side effects. Given its FDA approval and well-characterized pharmacokinetics, HALO could advance to clinical application more rapidly than novel compounds^1,2^. However, key translational factors must be addressed: dose optimization and delivery are crucial. The 0.5 mg/kg/day dose in mice is significantly lower than psychiatric doses in humans, suggesting that SMA-relevant effects may be achieved at subtherapeutic levels, minimizing potential side effects. In support of this, our results indicate that HALO at the tested regimens does not measurably alter DRD2 expression, arguing against DRD2-related off-target liability. Moreover, we demonstrated a disease-dependent effect in mice with SMA compared with WT animals, providing a solid basis for subsequent validation and translational optimization of dosing and delivery.

Further preclinical pharmacokinetics and alternative administration routes, for example, to avoid plasmatic peaks upon oral administration with slow-release methods (which may increase the risk of extrapyramidal side effects) should also be explored. Moreover, HALO’s dual mechanism — enhancing SMN and providing neuroprotection — positions it as a potential adjunct to existing SMA treatments. Future studies should assess synergistic effects with gene therapy or splicing modulators, particularly for patients with partial responses to standard therapies. Finally, although our findings support HALO’s safety at low doses, long-term assessments of behavioral and neurodevelopmental effects in pediatric models remain necessary before advancing to clinical trials.

Collectively, these findings support HALO’s progression toward early-phase clinical evaluation, prioritizing dose refinement, pharmacokinetics in larger preclinical models, and regulatory engagement for potential fast-track approval in rare diseases. Given its dual mechanism of action, HALO could serve as a complementary therapy for patients with residual motor deficits despite SMN-restoring treatments or as a stand-alone option in milder SMA cases. Future work should define human-equivalent dosing, biomarker-driven patient selection, and combination strategies to establish the optimal role of HALO in SMA treatment.

### Supplementary Information

Supplementary materials for this manuscript are available on the Experimental & Molecular Medicine website (http://www.nature.com/emm/).

## Supplementary information

Supplementary Information

Full unedited immunoblotting membranes

Supplementary Dataset 1

Supplementary Dataset 2

Supplementary Movie 1

## Data Availability

All data are available in the main text or Supplementary material. Supplementary Data files 1 and 2 provide complete RNA-seq quantification in spinal cord and quadriceps of treated delta 7 mice, respectively. The RNA-seq datasets generated during this study are publicly available in the NCBI BioProject repository under accession number PRJNA1251322 (https://www.ncbi.nlm.nih.gov/bioproject/?term=PRJNA1251322).
