# Supplementary material for: Haloperidol induces neuroprotection and enhances neuromuscular function in both murine and human models of spinal muscular atrophy
Source: Exp Mol Med. 2026 Apr 13;58(4):1216–29. doi: 10.1038/s12276-026-01689-0 (PMC13144737; doi:10.1038/s12276-026-01689-0)
Supplement: Supplementary file 1 — Supplementary Information [file 12276_2026_1689_MOESM1_ESM.pdf]

Supplementary Materials for:

“Haloperidol induces neuroprotection and enhances neuromuscular function in both murine and human models of Spinal Muscular Atrophy”

Giovanna Menduti *et al.*

Corresponding author: Giovanna Menduti, giovanna.menduti@unito.it

This file includes:

- Supplementary Materials and Methods
- References for Supplementary Materials and Methods
- Supplementary Fig. 1 to 11
- Supplementary Tables 1 to 6
- Legends for Supplementary Data Files 1 and 2, and Supplementary Movie 1

Supplementary Materials and Methods

Animal model and genotyping

For the *in vivo* HALO treatment, we used delta 7 mice (*SMN2+/+; SMN2Δ7+/+; Smn-/-*; Stock No. 005025; Jackson Lab, Bar Harbor, ME, USA) as severe SMA murine model, with early impairment of motor behavior correlated with motor neuron loss, and a lifespan of about 2 weeks [1]. The colony was maintained by interbreeding carrier mice, and the offspring were genotyped on tail DNA [extracted according to [2]] by PCR assays [3] at P0-1. Genotyping assessed the three possible genotypic variants of the Smn locus mice (*Smn+/+*, *Smn+/–* or *Smn-/-*), hence, for the subsequent experiments and analyses, we employed delta7 (*Smn-/-*) and WT (*Smn+/+*) mice. Pups were kept in cages with the dams, with free access to water and food, under a 12/12-h light/dark cycle. All efforts were made to minimize the number of animals used: for this purpose, behavioral, molecular, and histological results from some SMA control mice (VHL group, see below) were the same as those reported in [4]. Eighty-five delta7 SMA mice were used for behavioral, histological, molecular, and survival analyses. Moreover, 14 WT mice were used for weight analysis, immunofluorescence and/or western blot analysis.

HALO or VHL administration

SMA animals were injected daily, subcutaneously, with HALO (0.5 mg/kg, HY-14538, MedChemExpress, NJ, USA) or vehicle alone (VHL, 2% DMSO in 0.9% NaCl solution). The animals were treated from P2 to P12 (VHL n = 24; HALO n = 16) or until sacrifice for the survival analysis (VHL n = 33; HALO n = 12). In the latter case, animal sacrifices for humanitarian endpoints were applied when and if mice exhibited severe neurological/motor symptoms together with excessive weight loss (greater than 21±5% of the maximum peak weight achieved). A limited number of animals received HALO 0.25 mg/kg (WT VHL n = 6; WT HALO n =7).

Weight assessment and behavioral tests

From P2/P4 (depending on the test), both VHL-treated (n=21) and HALO-treated mice (HALO n=10) underwent daily assessment of weight and motor performance until P12, using a battery of behavioral motor tests, according to [5]. Briefly, the pups were moved to a dedicated behavioral testing room (under controlled conditions of light and noise) and maintained under infrared light, hence returned to maternal care after handling. The mice were first weighed and then underwent the following motor tests: tail suspension, righting reflex, hindlimb suspension, negative geotaxis tests (the

latter from P4). For the hindlimb suspension test, only the hindlimb posture was evaluated in the assigned score, according to [5].

Tissue collection and processing

At P12, mice were sacrificed depending on the following analyses. For the molecular assays (immunoblotting, Jess analysis and RNA seq analysis), treated SMA mice (VHL n=10, HALO n=13) and WT (n=4) were sacrificed by cervical dislocation: the whole spinal cord, brain and skeletal muscles (quadriceps and gastrocnemius), heart and liver were harvested, rapidly frozen in liquid nitrogen and then stored at -80°C until being processed. Moreover, for hematoxylin/eosin (H/E) staining, fresh quadriceps and gastrocnemius skeletal muscles were harvested, directly embedded in cryostat medium and frozen (Killik, Bio-Optica), and then cut into transverse 30 µm-thick slices: samples were collected directly on 4% gelatin-coated slides, air-dried overnight and then stored at -20°C until the staining.

For Nissl staining and immunohistochemical reactions, treated SMA mice (VHL n=5, HALO n=5) and WT (n=3) mice were anesthetized by gaseous anaesthesia and transcardially perfused with 4% buffered paraformaldehyde (PFA). Then, the lumbar spinal cords (L1-L5), quadriceps and gastrocnemius muscles were dissected, postfixed in 4% PFA for 2 h and incubated overnight in 30% sucrose in 0.1 M phosphate buffer solution. Next, the spinal cord samples were serially cut into transverse 40µm-thick sections, free-floating collected and stored in an antifreeze solution, while quadriceps and gastrocnemius samples were longitudinally cut in 30 µm-thick sections, directly collected onto 4% gelatin-coated slides, and air-dried overnight. All the samples were finally stored at -20°C.

Immunoblotting analysis in mice tissue samples and hiPSC-derived spinal MNs

For the analysis of the expression of SMN protein by western blot (WB), spinal cord and skeletal muscle (quadriceps and gastrocnemius) samples from treated delta7 and WT mice underwent to lysis, protein quantification, mechanical and chemical denaturation, and SDS-page and "semi-dry" transfer according to [4]. All membranes were incubated overnight with SMN- and in Vinculin-antibody solution [diluted in 2% nonfat dried milk in PBS-Tween 0.2%, (PBS-T)] at 4°C, under shaking; all antibodies' details and dilutions are listed in **Supplementary Table 1.** Next, HRP-conjugated secondary antibody solution (**Supplementary Table 1**), diluted in 2% nonfat dried milk in PBS-T, were incubated (1 h, RT, under shaking).

For all protein analysis, immunolabeling was detected with Clarity™ Western ECL Blotting Substrates (Bio-Rad) by using the ChemiDoc™ imaging system (Bio-Rad). All densitometric quantitation of bands intensity for different protein

expressions was calculated with reference to Vinculin protein levels (referred loading control), using the Fiji software (Image J, NIH) [6].

hiPSC-derived MNs treated or not, were lysed in RIPA 1X buffer (Sigma®) containing protease inhibitors (Sigma®) and phosphatase inhibitors (Roche®). Proteins were quantified by Pierce BCA Protein Assay kit (Pierce®) using a multiplate colorimetric reader, CLARIOstar (BMG Labtech®). Protein extracts were loaded on a 4%–12% SDS-PAGE gradient (NuPage Bis-Tris gels, Invitrogen®) and transferred onto Gel Transfer Stacks PVDF membranes (Invitrogen®) using the iBlot2 Dry Blotting System (Invitrogen®). Membranes were then incubated overnight at 4°C with the primary SMN or β-Actin antibodies in PBS-T + 5% milk (antibody and dilutions are listed in **Supplementary Table 1**). After hybridization with fluorescent secondary antibody, immunofluorescent bands were revealed and quantified on an Odyssey DLx Imaging System. Equal protein loading was verified by the detection of β-Actin

To perform DRD2 protein detection using the Jess™ Automated Western Blot system (Bio-techne), following the manufacturer's instructions (Bio-techne, Cat# DM-TP01, RP-001, SM-FL004, DM-001). Mice brain and hiPSC-derived MNs samples were homogenized and extracted in RIPA protease free buffer (Thermo Fisher Scientific #10230544; proteases inhibitors: Biotest #11697498001 and #4906837001), and quantified using BCA method (Thermo Fisher Scientific, Cat##10741395). 4µl of protein at 1,25mg/ml and 0,75mg/ml, respectively were loaded into the microfluidic capillary system. Protein separation, immunodetection, and chemiluminescent signal acquisition were carried out using the Compass for Simple Western software. Band intensity was quantified and normalized to Total Protein measures. The full, uncropped gels and blots are provided in the **Supplementary File 2**: " **Full unedited immunoblotting membranes**".

RNA massive sequencing

1. **RNA extraction, Sequencing and Preprocessing:** Total RNA was extracted from the spinal cord and quadriceps of SMA mice treated with HALO or VHL (spinal cord: HALO, n=4; VHL, n=5; quadriceps: HALO, n=4; VHL, n=4) using an RNeasy Kit for RNA Purification (QIAGEN #74104), and processed for RNA sequencing. Library preparation was performed using the Illumina TruSeq Stranded mRNA Kit, followed by paired-end sequencing (2 × 100 bp) on an Illumina NovaSeq 6000 S1 platform at a depth of 50 million reads per sample. Quality control of raw sequencing reads was conducted using Trimmomatic [7] to remove adapter sequences and low-quality bases. Reads were aligned to the reference genome (GRCh38) using STAR [8]with default parameters for splice-aware alignment.
2. **Differential Gene Expression Analysis:** Differential gene expression analysis was performed using the Bioconductor package edgeR [9]within the R statistical environment (v4.1.3). Raw read counts were normalized

using the trimmed mean of M-values (TMM) method. Data exploration, differential expression testing, and statistical adjustments were carried out following standard RNA-seq analysis workflows. The Benjamini-Hochberg method was applied for multiple testing correction, and differentially expressed genes ($p < 0.05$) were considered significant. SARTools was used for workflow optimization and statistical validation.

3. **Functional Enrichment Analysis:** Functional enrichment of differentially expressed genes was performed using Metascape (https://metascape.org) [10] for Gene Ontology (GO) network analysis and DAVID (https://davidbioinformatics.nih.gov) for Kyoto Encyclopedia of Genes and Genomes (KEGG) pathway analysis. Additional candidate genes associated with apoptosis, neuroinflammation, and other biological pathways were identified through the Molecular Signatures Database (MSigDB; www.gsea-msigdb.org) [11].
4. **Differential Alternative Splicing Analysis:** Alternative splicing (AS) events were analyzed using rMATS v4.1.2, identifying significant events (FDR < 0.05) across five AS types: skipped exon (SE), mutually exclusive exons (MXE), alternative 3' (A3SS) and 5' (A5SS) splice sites, and retained intron (RI). AS events were classified as annotated (fromGTF) or novel (fromGTF.novelJunction). Exon-level differential usage (DEU) was assessed using edgeR, identifying exons with significantly altered inclusion (FDR < 0.05).
5. **Differential Exon Usage Analysis:** Differential exon usage (DEU) was assessed using edgeR *(66)*. Exon-level count matrices were constructed, and log-fold changes (logFC) for individual exons were compared against the overall gene logFC. DEU was determined using F-tests, identifying exons with significantly different inclusion levels between treatment and control groups (FDR < 0.05). Output files included *contrast_TREATMENT_CONTROL.usage.exon.csv* (full DEU results) and *contrast_TREATMENT_CONTROL.usage.exon.sig005.xlsx* (significant exons at FDR < 0.05).
6. **SMN Target Gene Splicing Analysis:** AS events were cross-referenced with a curated list of SMN target genes from POSTAR3 [12]. Functional annotation was performed using MSigDB and analyzed with clusterProfiler [13], with significant enrichment defined as $p < 0.01$.
7. **Statistical and Computational Methods:** All analyses were conducted in R (v4.1.3), with data visualization via custom scripts. The Benjamini-Hochberg method controlled the false discovery rate.

Nissl staining and stereological counts of spinal MNs

For the Nissl staining, free-floating SMA spinal cord sections (VHL n=3, HALO n=3) were mounted on 4% gelatin-coated slides and stained as previously described *(15)*. The nucleoli of alpha MNs in the L1-L5 spinal cord were counted on serial sections (one every 320 μm) by using StereoInvestigator software (MicroBrightField Inc.). Only

neurons ≥ 80 μm^2 in area (i.e. alpha MNs) and located in a proper position in the ventral horns were included. A 15 -μm guard zone, a 100 μm × 100 μm counting frame size and a 150 μm × 150 μm scan grid size were set. The cell density (MN number/mm^3) was obtained by using Optical Fractionator stereological technique and a computer-assisted microscope (Nikon Eclipse E600 microscope), and the data analysis software NeuroExplorer (MicroBrightField). Representative images of spinal lumbar tract sections were acquired with Nikon Eclipse E600 microscope equipped with Optronics MicroFire digital camera.

Immunofluorescence staining and morphological analysis on spinal cord sections

Immunofluorescence stainings were performed as previously reported [4]. Briefly, sections were incubated overnight with primary antibody solutions at 4°C, under shaking (SMI32, Cleaved Caspase 3, GFAP and IBA1) (**Supplementary Table 1**), followed by incubation with proper fluorochrome-conjugated secondary antibody solution mixed with 4′, 6 Diamino-2 phenyindole Dilactate (DAPI; 1:200; D9564- 10MG, Sigma-Aldrich) (1 h 30 min, RT, under shaking). All antibodies' details and dilutions are listed in **Supplementary Table 1.** Finally, samples were washed and coverslipped with anti-fade mounting medium Mowiol. Confocal acquisitions were performed with a Leica TCS SP5 confocal laser scanning microscope (Leica Microsystems); unless otherwise stated, images were acquired with 40X oil objective (N.A.: 1.25), 0.5 μm z-step size, 14 μm z-volume, acquisition speed 100 Hz, with a resolution of 1024×1024 pixels. Imaging analyses were performed on maximum projections Z-stacks images by using FIJI software (ImageJ) [6]. The Cleaved Caspase 3-positive cell count and the astrogliosis semiquantitative analysis (based on GFAP-positive cell density) were performed by using the Fiji software according to [4]. Four animals were analysed for each group, four spinal cord slices were evaluated for each animal. Concerning the microglia cell phenotype characterization and classification, the confocal micrographs of ventral horn spinal cord were acquired with 40X oil objective (N.A.: 1.25) with 1.5X zoom, 0.5 μm z-step size, 20 μm z-volume, acquisition speed 200 Hz, with a resolution of 1024× 1024 pixels. The analysis of the number of microglia cells was achieved by manually counting IBA1-positive cells in thresholded images; overall, at least 4 sections were analysed for each mouse (VHL n=4, HALO n=4). Morphological analysis of microglia cells was performed by readapting protocols from [14,15]. Briefly, maximum projections Z-stacks images were first converted to 8-bit grayscale images and processed, sequentially, for the Unsharp Mask filter and the Despeckle tool. Then, the images were binarized, by applying an automatic threshold and processed with the "Close" and noise reduction tools. Next, binary images were analysed with the AnalyzeSkeleton (2D/3D) and for Frac lac plugins. All the resulting data from the skeletonization of microglial cell images were further filtered, then the values for the total length of the cell processes and endpoints were normalized to the number of microglia cell bodies in the corresponding image. At least 100 microglia cells per mouse were processed

from SMA (VHL n=4, HALO n=4) and WT (n=3) mice. Moreover, using the FracLac analysis plugin, the convex hull and bounding circle of randomly selected microglial cells were evaluated: in detail, a single binary cell in an image was converted into an outline (with the Outline tool) and then scanned into the FracLac box counting scan. Then, the fractal dimension, density, and span ratio data were obtained. At least 16 microglia cells per mouse have been processed for skeletonization analysis from SMA (VHL n=4, haloperidol n=4) and WT (n=3) mice. For all the Skeletonization and FracLac applications, the analysis parameters were set according to [14]. The different classification of microglial morphology (ramified/de-ramified&rod, de-ramified&rod and ramified) in treated SMA mice compared to WT controls was obtained by performing Pearson correlation analysis on the average cell branching, cell complexity, and shape data in each group, according to [15].

Immunofluorescence staining on skeletal muscle sections and NMJ analysis

Skeletal muscle longitudinal sections were stained for NMJ detection according to [4]. Neurofilament 145 kDa antibody and fluorescent α-bungarotoxin (BTX) conjugated with Alexafluor-555 (B35451, Invitrogen) details and dilutions are listed in **Supplementary Table 1**. For NMJ innervation analysis, the NF-positive fibers reaching each NMJ were assessed and counted with a computer-assisted microscope (Nikon Eclipse E600 microscope), allowing NMJ classification as mono-innervated, multi-innervated or denervated. At least 50 NMJs per animal were processed in both groups of SMA mice (VHL n=4, HALO n=4).

Histological analysis of skeletal muscle atrophy

Morphological analysis of the quadriceps and gastrocnemius sections were stained with H/E, according to [4]. For each animal (VHL n =4, HALO n=5), at least 100 fibers for each animal were drawn with Neurolucida software (MicroBrightField Inc.) and a computer-assisted microscope (Nikon Eclipse E600 microscope), allowing the morphological evaluation of mean fiber area, perimeter, minimum and maximum Feret's diameters. Subsequently, the resulting data were obtained by the associated NeuroExplorer data analysis software (MicroBrightField) and averaged. The resulting averages from individual animals were reported. Representative images of skeletal muscle fibers were acquired with a Nikon Eclipse E600 microscope equipped with Optronics MicroFire digital camera.

Human induced pluripotent stem cells

Human induced pluripotent stem cell (hiPSC) lines were generated by reprogramming Coriell Biorepository fibroblasts derived from non-affected and SMA-affected patients as previously described [4]. Three independent hiPSC clones were

generated from each line of healthy fibroblasts GM03814 and C03 as previously described [4]. Similarly, 3 independent clones of hiPSCs were generated from each fibroblast line issued from SMA type I (GM00232) and type II (GM03813) patients. Informed consents were obtained from all the patients included in this study, complying with the ethical guidelines of the institutions and with the legislation requirements. All the different hiPSC lines were grown on culture dishes coated with vitronectin (Gibco) and maintained in iPSBrew XF medium (Miltenyi Biotec) as previously described [4]. Cell passaging was performed manually every 5 days and culture medium was changed every 2 days.

Generation of spinal MNs from hiPSCs

The differentiation of hiPSC into spinal MNs was performed as previously described [4]. Briefly, hiPSCs were dissociated enzymatically using Stem Pro Accutase (ThermoFisher®) for 5 min at 37 °C, 5% CO2 and seeded in suspension in 25 cm2 flasks (Dutscher®) at 2 million per flask in an induced motoneuronal medium supplemented with different cytokines every 2 days. After 10 days of differentiation, the formed embryoid bodies were dissociated. During four supplemental days, MN progenitors matured into MNs, and MN phenotype was assessed by immunolabeling of Islet1 (**Supplementary Table 1**).

Co-culture of hiPSC-derived spinal MNs and human skeletal muscle cells

Human immortalized non-affected and SMA myoblasts were obtained from the MyoLine platform from the Institute of Myology and kindly provided by Vincent Mouly [16]. They were derived from patients and control biopsies provided by MyoBank, affiliated to EuroBioBank with the French ministry agreement ref AC-2019-3502. Skeletal muscle cells were obtained from a quadriceps muscle biopsy of a 5-day-old unaffected infant (CTL) [17]a paravertebral muscle biopsy of an 11-year-old infant patient suffering from SMA type I (KM432-7PV). Primary cells were isolated from skeletal muscle, expanded and differentiated as described previously [16]. Briefly, primary and immortalized myoblasts were cultured in a growth medium and cryopreserved. The percentage of myoblast cells was estimated by immunolabelling for Desmin. For co-culture, spinal MNs must be differentiated from hiPSCs as described previously, but the embryoid bodies are dissociated at day 14 of differentiation and frozen. 384 micropatterned-well plates were designed and provided by CYTOO SA [18]. For the co-culture the myoblasts were plated at a density of 5,500 cells per well in myoblast growth medium (DMEM/F12-glutamax supplemented with 20% foetal bovine serum (Sigma-Aldrich) and 0.1% penicillin–streptomycin (ThermoFisher) with Y-27632 (10 µM, Stemcell). After 2 days, the growth medium was replaced by the differentiation medium (DMEM/F12-glutamax supplemented with 2% horse serum; ThermoFisher). On day 4, the hiPSC-derived MNs were thawed and plated at a density of 8,000 cells per well in N2B27 medium supplemented by brain-

derived neurotrophic factor (BDNF, 10 ng/mL, Peprotech), glial-derived neurotrophic factor (GDNF, 10 ng/mL, Peprotech), N-[N-(3,5-Difuorophenacetyl)-l-alanyl]-S-phenylglycine t-butyl ester (DAPT, 10 µM, Tocris) and Y-27632 (10 µM, Stemcell). Cultures have been maintained up to 11 days corresponding to 4 days of myogenic differentiation and 7 days of co-culture with hiPSC-derived MNs.

Treatment of hiPSC-derived MNs

To assess the effects of HALO, MN progenitors were plated into poly-l-ornithine/laminin-treated 384-well plates at 3,000 cells per well in N2B27 medium. After 4 days of differentiation, "early" spinal MNs were treated with HALO using the Bravo Automated Liquid Handling Platform (Agilent Technologies). Cells were treated every 3 days for the next 10 days. A solution of 0.1% DMSO (MilliporeSigma) was used as a negative control. After 10 days, the cell viability was quantified. HALO (Cat. No.: HY-66011), Risdiplam (Cat. No.: HY-109101) and Nusinersen (Cat. No.: HY-112980), were all provided by MedChemExpress.

Immunostaining of hiPSC-derived MNs and co-cultures

Cells were fixed with 4% PFA (Electron Microscopy Sciences) for 5 min at room temperature (RT) and washed with PBS. Cells were then incubated overnight at 4°C with primary antibodies (listed in **Supplementary Table 1**): Tuj1 (neuronal marker), ISL1 (MN marker), MF20 (myotube marker), AchR (NMJ marker). The cells were washed 3 times in PBS buffer and incubated for 2 h at RT with appropriate fluorescent-labelled secondary antibodies and Hoechst (5 µg/mL). Image acquisitions were performed using the automated imaging Cell Insight CX7 HCS Platform (Cellomics Inc) using the 20X objective. Masks and algorithms were set up on the HCS Reader software for automated quantification of the percentage of cells stained for ISL1, compared to the total number of cells. The neuritic network was calculated as the area of Tuj1 staining using an algorithm developed in-house. Co-culture images were acquired using Spinning Disk microscopy (Zeiss) with the 20X objective and a z-stack plan. For quantification of the AChR clustering and neuritic network, each image taken was processed as a maximum projection and analysed using Fiji Software. The total area and mean size of AChR clusters were determined by an algorithm developed in-house.

Declaration of LLM tools technologies in the writing process

During the creation of this work, the authors occasionally used ChatGPT-4.0 to refine the language and enhance readability. The content was then thoroughly reviewed and revised by the authors, who take full responsibility for the final version.

References for Supplementary Materials and Methods

1 Le TT, Pham LT, Butchbach MER, Zhang HL, Monani UR, Coovert DD *et al.* SMNΔ7, the major product of the centromeric survival motor neuron (SMN2) gene, extends survival in mice with spinal muscular atrophy and associates with full-length SMN. *Hum Mol Genet* 2005; 14: 845–857.

2 Meeker ND, Hutchinson SA, Ho L, Trede NS. Benchmarks Method for isolation of PCR-ready genomic DNA from zebrafish tissues. 2007; 43: 4–6.

3 Valsecchi V, Boido M, De Amicis E, Piras A, Vercelli A. Expression of muscle-specific MiRNA 206 in the progression of disease in a murine SMA model. *PLoS One* 2015; 10: 1–17.

4 Januel C, Menduti G, Mamchaoui K, Martinat C, Artero R, Konieczny P *et al.* Moxifloxacin rescues SMA phenotypes in patient - derived cells and animal model. *Cellular and Molecular Life Sciences* 2022. doi:10.1007/s00018-022-04450-8.

5 El-Khodor BF, Edgar N, Chen A, Winberg ML, Joyce C, Brunner D *et al.* Identification of a battery of tests for drug candidate evaluation in the SMNΔ7 neonate model of spinal muscular atrophy. *Exp Neurol* 2008; 212: 29–43.

6 Schindelin J, Arganda-Carreras I, Frise E, Kaynig V, Longair M, Pietzsch T *et al.* Fiji: an open-source platform for biological-image analysis. *Nat Methods* 2012; 9: 676–682.

7 Bolger AM, Lohse M, Usadel B. Trimmomatic: a flexible trimmer for Illumina sequence data. *Bioinformatics* 2014; 30: 2114–2120.

8 Dobin A, Davis CA, Schlesinger F, Drenkow J, Zaleski C, Jha S *et al.* STAR: ultrafast universal RNA-seq aligner. *Bioinformatics* 2013; 29: 15–21.

9 Robinson MD, McCarthy DJ, Smyth GK. edgeR: a Bioconductor package for differential expression analysis of digital gene expression data. *Bioinformatics* 2010; 26: 139–140.

10 Zhou Y, Zhou B, Pache L, Chang M, Khodabakhshi AH, Tanaseichuk O *et al.* Metascape provides a biologist-oriented resource for the analysis of systems-level datasets. *Nat Commun* 2019; 10: 1523.

11 Liberzon A, Birger C, Thorvaldsdóttir H, Ghandi M, Mesirov JP, Tamayo P. The Molecular Signatures Database (MSigDB) hallmark gene set collection. *Cell Syst* 2015; 1: 417–425.

12 Zhao W, Zhang S, Zhu Y, Xi X, Bao P, Ma Z *et al.* POSTAR3: an updated platform for exploring post-transcriptional regulation coordinated by RNA-binding proteins. *Nucleic Acids Res* 2022; 50: D287–D294.

13 Yu G, Wang L-G, Han Y, He Q-Y. clusterProfiler: an R package for comparing biological themes among gene clusters. *OMICS* 2012; 16: 284–287.

14 Young K, Morrison H. Quantifying Microglia Morphology from Photomicrographs of Immunohistochemistry Prepared Tissue Using ImageJ. *J Vis Exp* 2018. doi:10.3791/57648.

15 Morrison H, Young K, Qureshi M, Rowe RK, Lifshitz J. Quantitative microglia analyses reveal diverse morphologic responses in the rat cortex after diffuse brain injury. *Sci Rep* 2017; 7: 13211.

16 Chaouch S, Mouly V, Goyenvalle A, Vulin A, Mamchaoui K, Negroni E *et al.* Immortalized skin fibroblasts expressing conditional MyoD as a renewable and reliable source of converted human muscle cells to assess therapeutic strategies for muscular dystrophies: validation of an exon-skipping approach to restore dystrophin in Duche. *Hum Gene Ther* 2009; 20: 784–790.

17 Edom F, Mouly V, Barbet JP, Fiszman MY, Butler-Browne GS. Clones of human satellite cells can express in vitro both fast and slow myosin heavy chains. *Dev Biol* 1994; 164: 219–229.

18 Young J, Margaron Y, Fernandes M, Duchemin-Pelletier E, Michaud J, Flaender M *et al.* MyoScreen, a High-Throughput Phenotypic Screening Platform Enabling Muscle Drug Discovery. *SLAS Discov* 2018; 23: 790–806.

Supplementary Figures 1 to 11

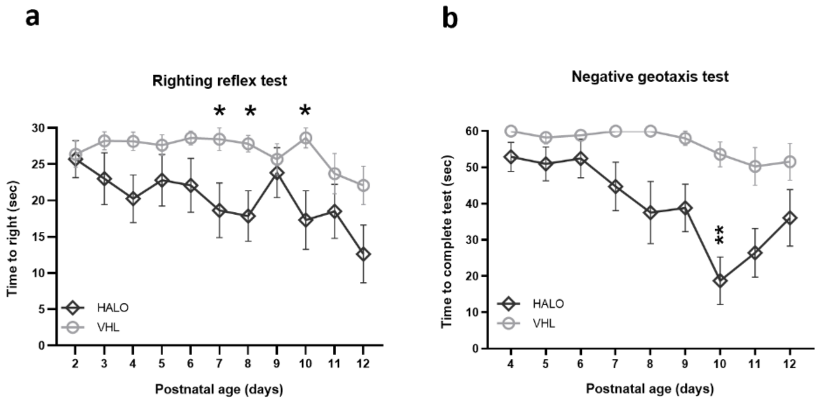

Supplementary Fig. 1. Haloperidol treatment improves the time of delta 7 mice to complete behavioral motor tests. Righting reflex (**a**) and negative geotaxis (**b**) of treated delta 7 mice. Data are expressed as mean± SEM, VHL n= 21, HALO n= 10; mixed-effects model with Geisser-Greenhouse correction, followed by Sidak's multiple comparison post hoc test, *p < 0.05, **p <0.01

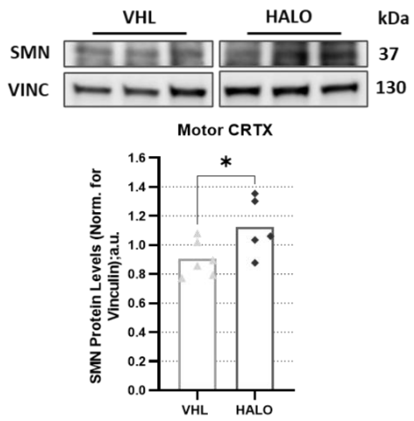

Supplementary Fig. 2. Effect of HALO on SMN protein levels in the motor cortex of SMA mice. Representative densitometry (up) and quantification (bottom) of SMN protein levels in VHL and HALO treated SMA motor cortex. Vinculin (VINC) protein levels were referred as loading control; data are shown as mean from the individual animals, VHL n= 6, HALO n= 5, Student's t-test, *$p <$ 0.05

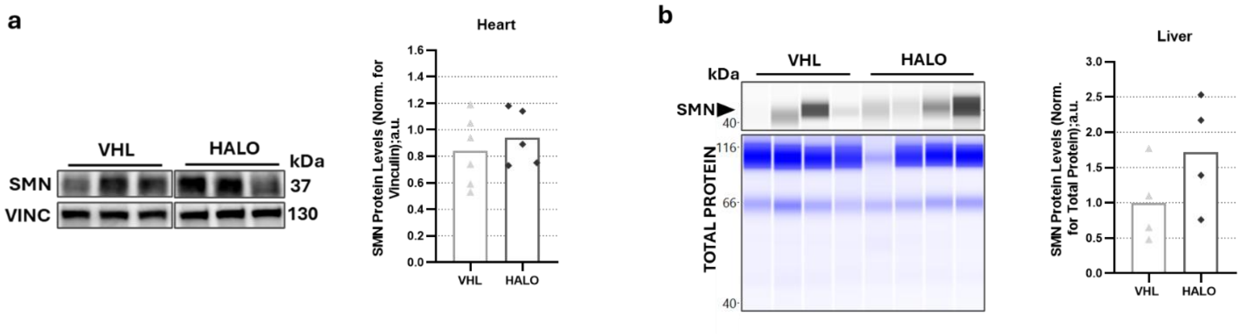

Supplementary Fig. 3. **Effect of HALO on SMN protein levels in SMN in the heart and liver of SMA mice.** Representative densitometry (left) and quantification (right) of SMN protein levels in VHL and HALO 0.5 mg/kg treated delta 7 heart (**a**) and liver (**b**). Vinculin or Total Protein levels were referred as loading control (in **a** and **b**, respectively); data are shown as mean from the individual animals, for heart analysis: VHL n= 6, HALO n= 5; for liver analysis: VHL n= 4, HALO n= 4.

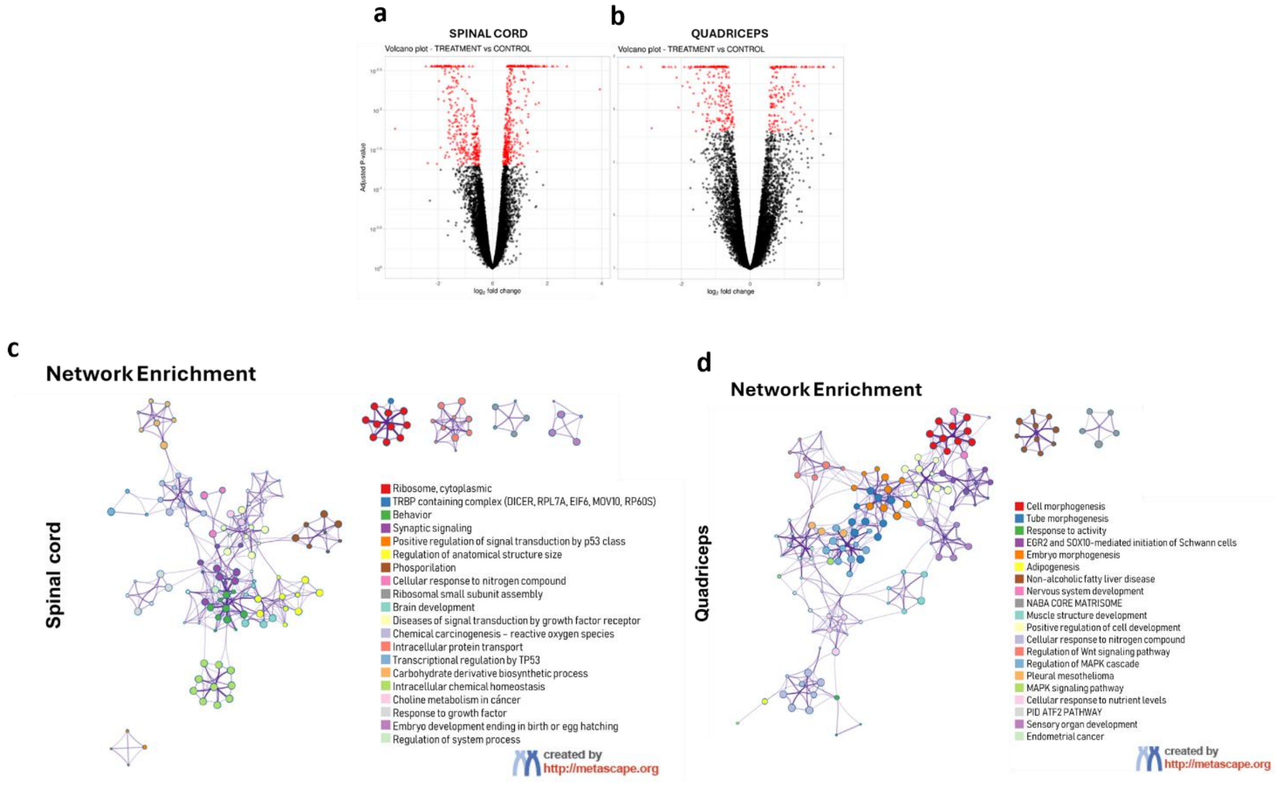

Supplementary Fig. 4. RNAseq results overview. a-b Vulcano plots for spinal cord (**a**) and quadriceps (**b**), showing in red genes that are significantly regulated. **c-d** enrichment map of commonly enriched ontology terms in RNA-Seq differentially regulated entities for spinal cord (**c**) and quadriceps (**d**). Color coding identifies the different clusters of enriched terms

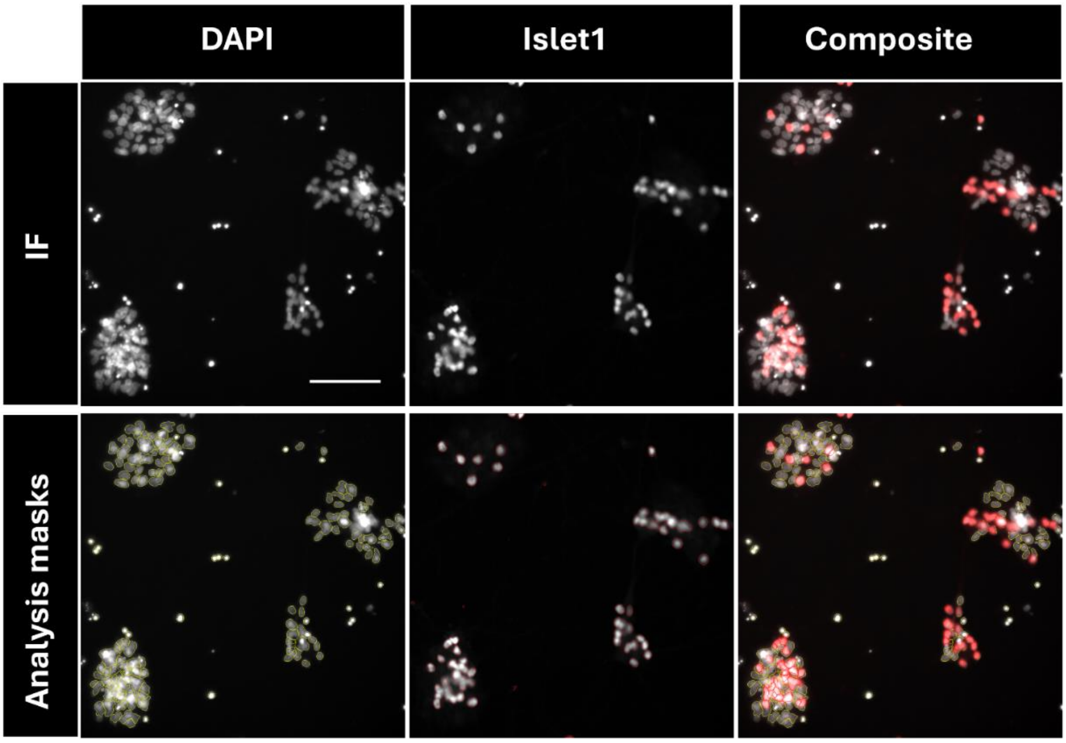

Supplementary Fig. 5. Image analysis of hiPSC derived MNs. Top: Representative images of an immunostaining of MNs at day 24. From left to right: DAPI staining in nuclei, Islet1 immunofluorescence, composite of bith channels. Scale bar: 100 µm. Bottom: corresponding analysis masks used to quantify the number of nuclei and the pourcentage of Islet1 positive cells

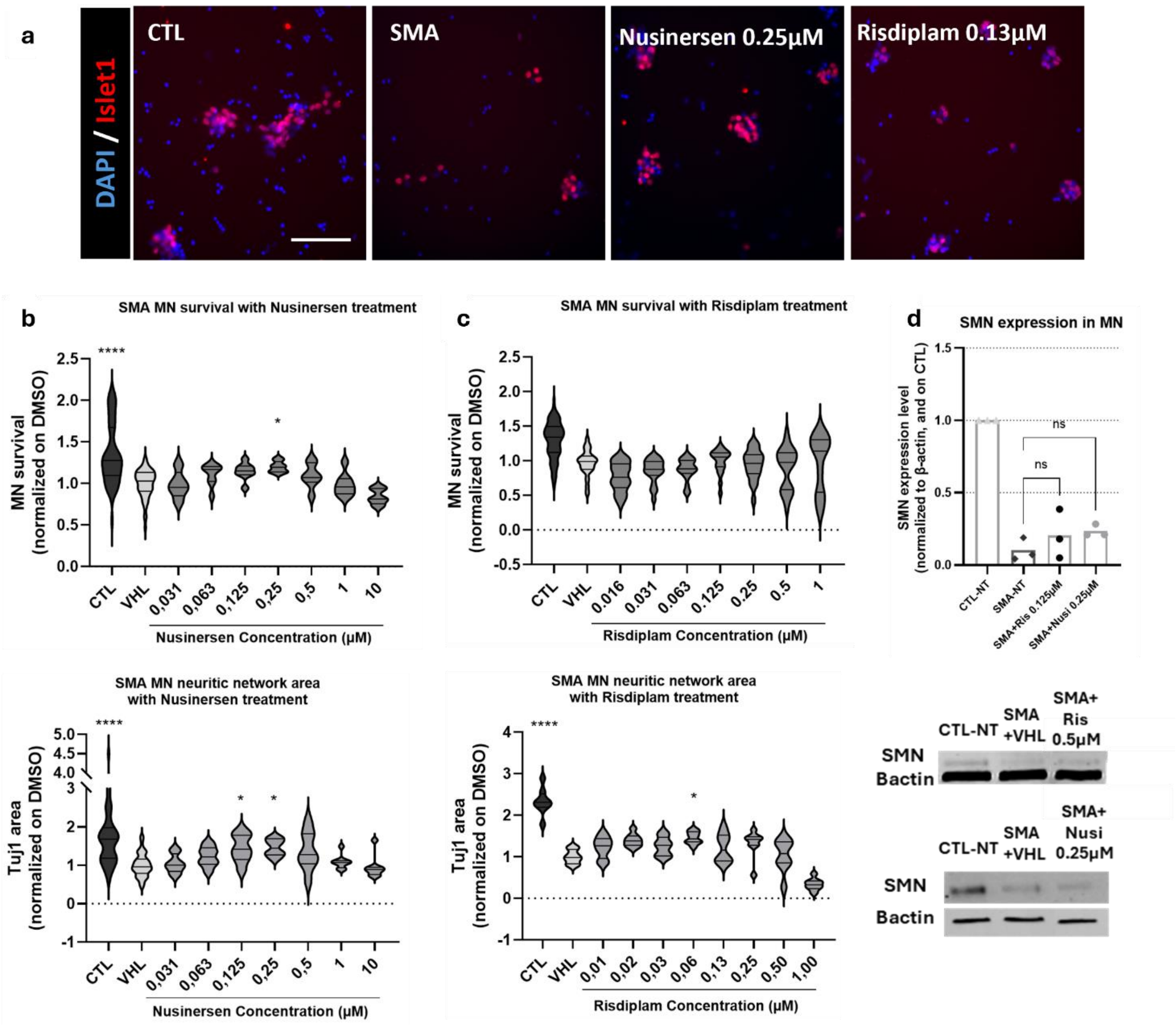

Supplementary Fig. 6. Effect of Risdiplam and Nusinersen on SMN expression and MN survival in SMA patient-derived iPSCs. **a** Immunostaining of MNs at day 24. From left to right: CTL (control) MNs; SMA MNs receiving vehicle (VHL); SMA MNs treated with Nusinersen at 0.25 µM; or Risdiplam at 0.13µM. Scale bar: 100 µm. **b** Quantification of MN survival (top) and neuritic network area (Tuj1-positive, bottom) after treatment with different doses of Nusinersen. Data are represented as violin plots of four independent experiments with four technical replicates each. **c** Quantification of MN survival (top) and neuritic network area (Tuj1-positive, bottom) after treatment with different doses of Risdiplam. Data are represented as violin plots of four independent experiments with four technical replicates each. **d** Representative densitometry (bottom) and quantification (up) of SMN proteins by Western blot. Data are displayed as the average SMN proteins, normalized to β-Actin and on CTL not treated (NT) cells, measured by three independent experiments, indicated by dots. All statistics were calculated using Kruskal-Wallis Dunn's multiple comparisons tests (ns: Not significant, *p > 0.05, **p < 0.01, ***p < 0.001, ****p < 0.0001)

Gene id	Chr	Start	End	Exon	Length	FC	P.Value	FDR
SMN2	chr5	70066934	70067134	exon 4	201	1,236	0,345	0,989
SMN2	chr5	70067294	70067446	exon 5	153	1,276	0,190	0,989
SMN2	chr5	70069235	70069330	exon 6	96	1,021	0,912	0,989
SMN2	chr5	70070641	70070751	exon 7	111	1,040	0,817	0,989
SMN2	chr5	70076521	70076574	exon 8	54	1,407	0,058	0,989
SMN2	chr5	70077019	70077595	3'UTR	577	0,925	0,620	0,989

Supplementary Fig. 7. SMN splicing events in spinal cord of VHL- vs HALO-treated SMA mice. *SMN2* splicing data were obtained from the spinal cord of VHL-treated (n = 5) and HALO-treated (n = 4) mice. Exons were identified based on genomic coordinates and exon lengths using the Ensembl database.

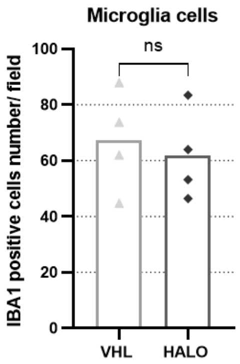

Supplementary Fig. 8. IBA1-Positive microglial cell counts are unchanged in HALO-treated mice compared to VHL controls. Summary data of microglia cell number in VHL and HALO treated mice, data are expressed as mean cell number per individual animal; n= 4 per group, Student's t-test.

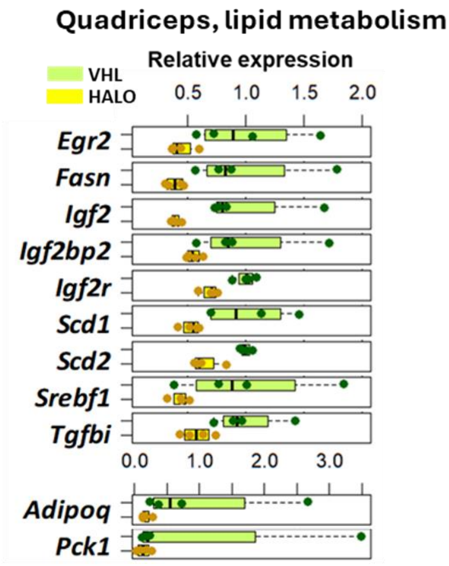

Supplementary Fig. 9. RNA-seq data from quadriceps indicate structural and metabolic changes linked to improved skeletal muscle function. Genes show values normalized to VHL values average and present significant differences between treated and VHL samples, according to adjusted p-value < 0.05.

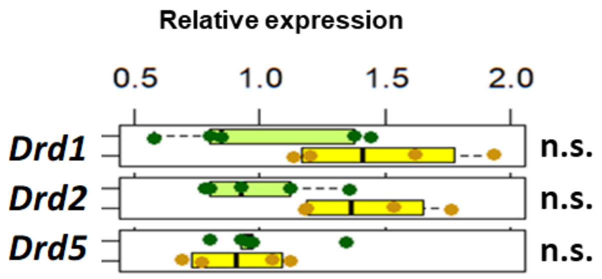

Supplementary Fig. 10. Dopamine receptors expression in spinal cord. RNA-seq data for Drd family members were obtained from the spinal cord of VHL (n = 5) and HALO-treated (n = 4) mice. No significant treatment-related changes were observed.

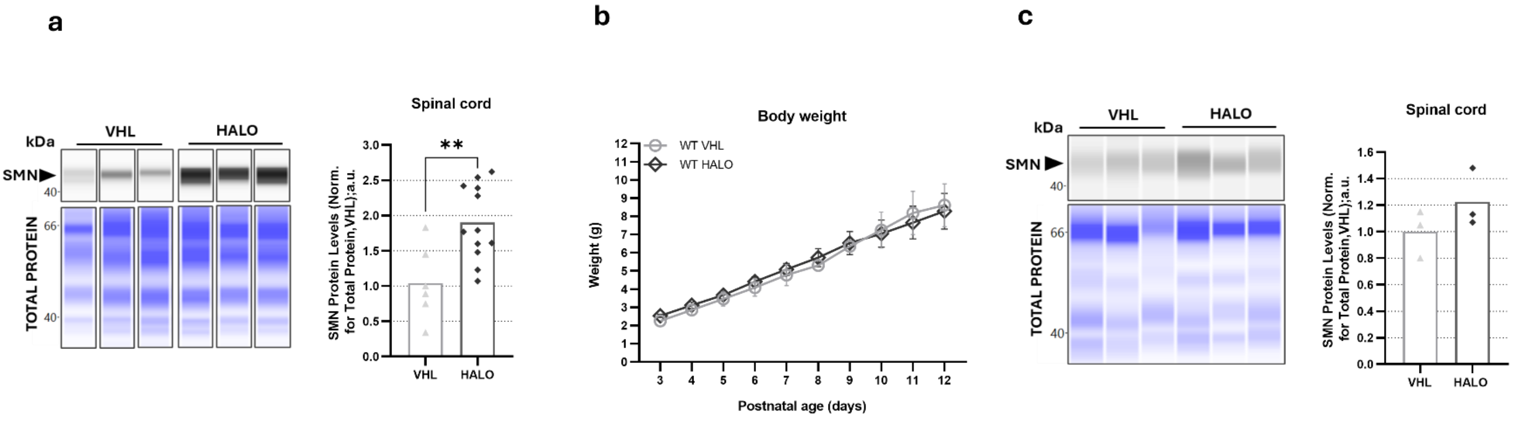

Supplementary Fig. 11. **Disease-dependent effects of low-dose HALO in SMA and WT mice (0.25 mg/kg). a** Representative densitometry (left) and quantification (right) of SMN protein levels in VHL and HALO 0.25 mg/kg treated delta 7 spinal cord. Total Protein levels were referred as loading control; data are shown as mean from the individual animals, VHL n= 7, HALO n= 12, Student's t-test, $^{**}p < 0.01$. **b** Body weight assessment in WT mice treated with 0.25 mg/kg Haloperidol (WT HALO) compared to WT mice treated with vehicle (WT VHL) from postnatal day 3 (P3) to P12. WT VHL n= 6, WT HALO n= 7. **c** Representative densitometry (left) and quantification (right) of SMN protein levels in VHL and HALO 0.25 mg/kg treated WT spinal cord. Total Protein levels were referred as loading control; data are shown as mean from the individual animals, VHL n= 4, HALO n= 5, Student's t-test, $^{*}p < 0.05$.

Supplementary Tables 1 to 6

Supplementary Table 1. List of primary and secondary antibodies used in immunofluorescence and immunoblotting experiments.

Model	Antibodies	IF/WB	Designation	Reference	Host	Provider	Dilution
SMA delta 7 mice	Primary	IF	Neurofilament H (NF-H) SMI32	801702	Mouse	BioLegend	1:1,000
			Cleaved Caspase 3 (Asp175)	9661	Rabbit	Cell Signaling Technology	1:400
			Glial Fibrillary Acidic Protein (GFAP)	Z0334	Rabbit	DAKO Cytomation	1:500
			IBA1	019-19-741	Rabbit	WAKO	1:1,000
			Neurofilament clone 2H3C	AB2314897	Mouse	DSHB	1:200
	Fluorescent conjugated	IF	α-bungarotoxin (BTX) conjugated with Alexafluor-555	B35451		Invitrogen	1:500
		WB	SMN	610646	Mouse	BD Transduction	1:2,000
			Total OXPHOS Rodent WB Antibody Cocktail	AB110413	Mouse	Abcam	1:1,000
			Monoclonal anti-Vinculin, clone HVIN-1	V9131-100UL	Mouse	Merck	1:2,000
			DRD2	ab130295	Rabbit	Abcam	1:1000
		JESS	DRD2	AB5084P	Rabbit	Merck	1:25
	Secondary	IF	Cy™2 AffiniPure Donkey Anti-Mouse IgG (H +L)	AB_2340826		Jackson ImmunoResearch	1:400
			Cy™2 AffiniPure Donkey Anti-Rabbit IgG (H + L)	AB_2340612		Jackson ImmunoResearch	1:400
			Cy™3 AffiniPure Donkey Anti-Mouse IgG (H + L)	AB_2340813		Jackson ImmunoResearch	1:400
			Cy™3 AffiniPure Donkey Anti-Rabbit IgG (H + L)	AB_2307443		Jackson ImmunoResearch	1:400
		WB	GOAT anti-mouse IGG HRP	1706516		Biorad	1:10,000
			GOAT anti-rabbit IGG HRP	1706515		Biorad	1:10,000
hiPCS-derived MNs	Primary	IF	Islet 1 (ISL1)	AF1837	Goat	R&Dsystems	1:500
			Tubulin β III (Tuj1)	802001	Rabbit	Biolegend	1:1000

			Myosin Heavy Chain (MF20)	MF 20	Mouse	DSHB	1:200
			Acetylcholine receptor (AchR)	mAB 35	Rat	DSHB	1:350
		WB	SMN	610647	Mouse	BD Transduction	1:1,000
			Anti-β-Actin	926-42210	Rabbit	Li-cor®	1:1,000
			Anti-β-Actin	926-42212	Mouse	Li-cor®	1:1,000
	Secondary	IF					
			Donkey anti-goat A647	A21447	-Donkey	ThermoFisher	1:1000
			Donkey anti-rabbit A555	A31572	Donkey	ThermoFisher	1:1000
		WB	IRDye 800CW Donkey anti-Mouse	926-32212	Donkey	Li-cor®	1:15,000
			IRDye 680RD Donkey anti-Rabbit	926-68073	Donkey	Li-cor®	1:15,000

Supplementary Table 2. List of genes (in alphabetic order) differentially expressed in spinal cord in response to HALO according to RNASeq analysis; used in Figures 2, 5 and 6.

Gene name	Description	Ensembl Id
Adam10	A disintegrin and metallopeptidase domain 10	ENSMUSG00000054693
Adam22	A disintegrin and metallopeptidase domain 22	ENSMUSG00000040537
Ager	Advanced glycosylation end product-specific receptor	ENSMUSG00000015452
Atf2	Activating transcription factor 2	ENSMUSG00000027104
Atm	Ataxia telangiectasia mutated	ENSMUSG00000034218
Cacna1e	Calcium channel, voltage-dependent, R type, alpha 1E subunit	ENSMUSG00000004110
Chrm2	Cholinergic receptor, muscarinic 2, cardiac	ENSMUSG00000045613
Chrm5	Cholinergic receptor, muscarinic 5	ENSMUSG00000074939
Chrna7	Cholinergic receptor, nicotinic, alpha polypeptide 7	ENSMUSG00000030525
Cib2	Calcium and integrin binding family member 2	ENSMUSG00000037493
Clcn5	Chloride channel, voltage-sensitive 5	ENSMUSG00000004317
Dgkb	Diacylglycerol kinase, beta	ENSMUSG00000036095
Dlg2	Discs large MAGUK scaffold protein 2	ENSMUSG00000052572
Erbb4	Erb-b2 receptor tyrosine kinase 4	ENSMUSG00000062209
Fmr1	Fragile X messenger ribonucleoprotein 1	ENSMUSG00000000838
Gabpa	GA repeat binding protein, alpha	ENSMUSG00000008976
Gabrb2	Gamma-aminobutyric acid (GABA) A receptor, subunit beta 2	ENSMUSG00000007653
Gabrb3	Gamma-aminobutyric acid (GABA) A receptor, subunit beta 3	ENSMUSG00000033676
Gabrg1	Gamma-aminobutyric acid (GABA) A receptor, subunit gamma 1	ENSMUSG00000001260
Gpr158	G protein-coupled receptor 158	ENSMUSG00000045967
Grin2a	Glutamate receptor, ionotropic, NMDA2A (epsilon 1)	ENSMUSG00000059003
Grm5	Glutamate receptor, metabotropic 5	ENSMUSG00000049583
Htr2a	5-hydroxytryptamine (serotonin) receptor 2A	ENSMUSG00000034997
Kcnc2	Potassium voltage gated channel, Shaw-related subfamily, member 2	ENSMUSG00000035681
Kcnd3	Potassium voltage-gated channel, Shal-related family, member 3	ENSMUSG00000040896
Kcnj6	Potassium inwardly-rectifying channel, subfamily J, member 6	ENSMUSG00000043301
Kcnma1	Potassium large conductance calcium-activated channel, subfamily M, alpha member 1	ENSMUSG00000063142
Lrrtm2	Leucine rich repeat transmembrane neuronal 2	ENSMUSG00000071862
Myo5a	Myosin VA	ENSMUSG00000034593
Neto1	Neuropilin (NRP) and tolloid (TLL)-like 1	ENSMUSG00000050321
Nr3c2	Nuclear receptor subfamily 3, group C, member 2	ENSMUSG00000031618
Nrcam	Neuronal cell adhesion molecule	ENSMUSG00000020598
Pdcd5	Programmed cell death 5	ENSMUSG00000030417
Pten	Phosphatase and tensin homolog	ENSMUSG00000013663
Rock2	Rho-associated coiled-coil containing protein kinase 2	ENSMUSG00000020580
Xiap	X-linked inhibitor of apoptosis	ENSMUSG00000025860

Supplementary Table 3. List of genes (in alphabetic order) with significant alternative splicing (p<0.05, FDR<0.05) in response to HALO according to RNASeq analysis; used in Figure 4.

Gene name	Description	Ensembl Id	Alternative splicing events p<0.05, FDR<0.05
Afdn	Afadin, adherens junction formation factor	ENSMUSG00000068036	14
Ankrd17	Ankyrin repeat domain 17	ENSMUSG00000055204	18
Apbb2	Amyloid beta (A4) precursor protein-binding, family B, member 2	ENSMUSG00000029207	4
Arap2	ArfGAP with RhoGAP domain, ankyrin repeat and PH domain 2	ENSMUSG00000037999	9
Arhgap32	Rho GTPase activating protein 32	ENSMUSG00000041444	1
Arih1	Ariadne RBR E3 ubiquitin protein ligase 1	ENSMUSG00000025234	2
Atp13a3	ATPase type 13A3	ENSMUSG00000022533	19
Atxn2	Ataxin 2	ENSMUSG00000042605	5
Bcat1	Branched chain aminotransferase 1, cytosolic	ENSMUSG00000030268	1
Calm1	Calmodulin 1	ENSMUSG00000001175	2
Cbx5	Chromobox 5	ENSMUSG00000009575	1
Ccdc50	Coiled-coil domain containing 50	ENSMUSG00000038127	2
Cct8	Chaperonin containing Tcp1, subunit 8 (theta)	ENSMUSG00000025613	1
Cd164	CD164 antigen	ENSMUSG00000019818	1
Chd3	Chromodomain helicase DNA binding protein 3	ENSMUSG00000018474	4
Ckap5	Cytoskeleton associated protein 5	ENSMUSG00000040549	23
Cnbp	Cellular nucleic acid binding protein	ENSMUSG00000030057	1
Cog4	Component of oligomeric golgi complex 4	ENSMUSG00000031753	1
Cpsf6	Cleavage and polyadenylation specific factor 6	ENSMUSG00000055531	1
Crnkl1	Crooked neck pre-mRNA splicing factor 1	ENSMUSG00000001767	3
Cyth2	Cytohesin 2	ENSMUSG00000003269	2
Ddx17	DEAD box helicase 17	ENSMUSG00000055065	11
Dhx15	DEAH (Asp-Glu-Ala-His) box polypeptide 15	ENSMUSG00000029169	1
Dicer1	Dicer 1, ribonuclease type III	ENSMUSG00000041415	1
Dido1	Death inducer-obliterator 1	ENSMUSG00000038914	2
Dnajc11	DnaJ heat shock protein family (Hsp40) member C11	ENSMUSG00000039768	1
Dock3	Dedicator of cyto-kinesis 3	ENSMUSG00000039716	3
Dock9	Dedicator of cytokinesis 9	ENSMUSG00000025558	1
Dst	Dystonin	ENSMUSG00000026131	5
Dync1h1	Dynein cytoplasmic 1 heavy chain 1	ENSMUSG00000018707	2
Eef1d	Eukaryotic translation elongation factor 1 delta	ENSMUSG00000055762	1
Eif3a	Eukaryotic translation initiation factor 3, subunit A	ENSMUSG00000024991	10
Eif4a2	Eukaryotic translation initiation factor 4A2	ENSMUSG00000022884	3
Eif5	Eukaryotic translation initiation factor 5	ENSMUSG00000021282	2
Enah	ENAH actin regulator	ENSMUSG00000022995	3
Eno1	Enolase 1, alpha non-neuron	ENSMUSG00000063524	1
Eprs	Glutamyl-prolyl-tRNA synthetase	ENSMUSG00000026615	11
Ext1	Exostosin glycosyltransferase 1	ENSMUSG00000061731	2
Fbxo11	F-box protein 11	ENSMUSG00000005371	1
Fxr2	FMR1 autosomal homolog 2	ENSMUSG00000018765	1

Galnt7	Polypeptide N-acetylgalactosaminyltransferase 7	ENSMUSG00000031608	2
Gbf1	Golgi-specific brefeldin A-resistance factor 1	ENSMUSG00000025224	1
Gcn1	GCN1 activator of EIF2AK4	ENSMUSG00000041638	2
Gls	Glutaminase	ENSMUSG00000026103	6
Gnas	GNAS (guanine nucleotide binding protein, alpha stimulating) complex locus	ENSMUSG00000027523	2
Gpi1	Glucose-6-phosphate isomerase 1	ENSMUSG00000036427	3
Gpm6b	Glycoprotein m6b	ENSMUSG00000031342	2
Grk2	G protein-coupled receptor kinase 2	ENSMUSG00000024858	1
Hnrnpd	Heterogeneous nuclear ribonucleoprotein D	ENSMUSG00000000568	1
Hnrnph1	Heterogeneous nuclear ribonucleoprotein H1	ENSMUSG00000007850	4
Hnrnpu	Heterogeneous nuclear ribonucleoprotein U	ENSMUSG00000039630	1
Hsp90aa1	Heat shock protein 90, alpha (cytosolic), class A member 1	ENSMUSG00000021270	5
Hsp90b1	Heat shock protein 90, beta (Grp94), member 1	ENSMUSG00000020048	1
Ikbip	IKBKB interacting protein	ENSMUSG00000019975	1
Ireb2	Iron responsive element binding protein 2	ENSMUSG00000032293	11
Kdm2a	Lysine (K)-specific demethylase 2A	ENSMUSG00000054611	2
Larp4	La ribonucleoprotein 4	ENSMUSG00000023025	10
Limd1	LIM domains containing 1	ENSMUSG00000025239	1
Lrp1	Low density lipoprotein receptor-related protein 1	ENSMUSG00000040249	1
Lrrfip1	Leucine rich repeat (in FLII) interacting protein 1	ENSMUSG00000026305	4
Man2c1	Mannosidase, alpha, class 2C, member 1	ENSMUSG00000032295	1
Map1b	Microtubule-associated protein 1B	ENSMUSG00000052727	3
Map4k4	Mitogen-activated protein kinase 4	ENSMUSG00000026074	1
Mdn1	Midasin AAA ATPase 1	ENSMUSG00000058006	1
Mga	MAX gene associated	ENSMUSG00000033943	3
Mib1	MIB E3 ubiquitin protein ligase 1	ENSMUSG00000024294	1
Mllt10	Myeloid/lymphoid or mixed-lineage leukemia; translocated to, 10	ENSMUSG00000026743	1
Mtrex	Mtr4 exosome RNA helicase	ENSMUSG00000016018	4
Mysm1	Myb-like, SWIRM and MPN domains 1	ENSMUSG00000062627	3
N4bp2l2	NEDD4 binding protein 2-like 2	ENSMUSG00000029655	1
Nav2	Neuron navigator 2	ENSMUSG00000052512	2
Ncbp3	Nuclear cap binding subunit 3	ENSMUSG00000020783	1
Ncor1	Nuclear receptor co-repressor 1	ENSMUSG00000018501	13
Nemf	Nuclear export mediator factor	ENSMUSG00000020982	2
Nfkb1	Nuclear factor of kappa light polypeptide gene enhancer in B cells 1, p105	ENSMUSG00000028163	1
Npepps	Aminopeptidase puromycin sensitive	ENSMUSG00000001441	13
Nrp1	Neuropilin 1	ENSMUSG00000025810	2
Nsd2	Nuclear receptor binding SET domain protein 2	ENSMUSG00000057406	1
Palld	Palladin, cytoskeletal associated protein	ENSMUSG00000058056	1
Pcm1	Pericentriolar material 1	ENSMUSG00000031592	12
Pfas	Phosphoribosylformylglycinamidine synthase (FGAR amidotransferase)	ENSMUSG00000020899	1
Phkb	Phosphorylase kinase beta	ENSMUSG00000036879	5
Phldb2	Pleckstrin homology like domain, family B, member 2	ENSMUSG00000033149	2
Pid1	Phosphotyrosine interaction domain containing 1	ENSMUSG00000045658	1
Plod2	Procollagen lysine, 2-oxoglutarate 5-dioxygenase 2	ENSMUSG00000032374	1

Pomp	Proteasome maturation protein	ENSMUSG00000029649	1
Ppp4r3b	Protein phosphatase 4 regulatory subunit 3B	ENSMUSG00000020463	2
Ppp6r3	Protein phosphatase 6, regulatory subunit 3	ENSMUSG00000024908	1
Prkn	Parkin RBR E3 ubiquitin protein ligase	ENSMUSG00000023826	2
Psap	Prosaposin	ENSMUSG00000004207	1
Ranbp2	RAN binding protein 2	ENSMUSG00000003226	5
Rangap1	RAN GTPase activating protein 1	ENSMUSG00000022391	1
Rbbp6	Retinoblastoma binding protein 6, ubiquitin ligase	ENSMUSG00000030779	2
Rbm25	RNA binding motif protein 25	ENSMUSG00000010608	1
Rbm6	RNA binding motif protein 6	ENSMUSG00000032582	3
Rdx	Radixin	ENSMUSG00000032050	1
Reps2	RALBP1 associated Eps domain containing protein 2	ENSMUSG00000040855	5
Rlf	Rearranged L-myc fusion sequence	ENSMUSG00000049878	8
Robo2	Roundabout guidance receptor 2	ENSMUSG00000052516	2
Rtn4	Reticulon 4	ENSMUSG00000020458	4
Samd4	Sterile alpha motif domain containing 4	ENSMUSG00000021838	1
Sdk1	Sidekick cell adhesion molecule 1	ENSMUSG00000039683	1
Sf1	Splicing factor 1	ENSMUSG00000024949	6
Sf3a1	Splicing factor 3a, subunit 1	ENSMUSG00000002129	1
Sfpq	Splicing factor proline/glutamine rich (polypyrimidine tract binding protein associated)	ENSMUSG00000028820	2
Slc20a1	Solute carrier family 20, member 1	ENSMUSG00000027397	2
Smarca5	SWI/SNF related, matrix associated, actin dependent regulator of chromatin, subfamily a, member 5	ENSMUSG00000031715	10
Soga1	Suppressor of glucose, autophagy associated 1	ENSMUSG00000055485	1
Son	Son DNA binding protein	ENSMUSG00000022961	7
Sptbn1	Spectrin beta, non-erythrocytic 1	ENSMUSG00000020315	1
Srrm2	Serine/arginine repetitive matrix 2	ENSMUSG00000039218	1
Srsf11	Serine and arginine-rich splicing factor 11	ENSMUSG00000055436	10
Srsf3	Serine and arginine-rich splicing factor 3	ENSMUSG00000071172	1
Sun1	Sad1 and UNC84 domain containing 1	ENSMUSG00000036817	1
Tanc1	Tetratricopeptide repeat, ankyrin repeat and coiled-coil containing 1	ENSMUSG00000035168	2
Tcerg1	Transcription elongation regulator 1 (CA150)	ENSMUSG00000024498	3
Tead1	TEA domain family member 1	ENSMUSG00000055320	2
Tia1	Cytotoxic granule-associated RNA binding protein 1	ENSMUSG00000071337	3
Tnrc18	Trinucleotide repeat containing 18	ENSMUSG00000039477	1
Tnrc6a	Trinucleotide repeat containing 6a	ENSMUSG00000052707	2
Top2a	Topoisomerase (DNA) II alpha	ENSMUSG00000020914	2
Tpm1	Tropomyosin 1, alpha	ENSMUSG00000032366	1
Tra2b	Transformer 2 beta	ENSMUSG00000022858	1
Tsc22d1	TSC22 domain family, member 1	ENSMUSG00000022010	1
Tuba1b	Tubulin, alpha 1B	ENSMUSG00000023004	1
Tug1	Taurine upregulated gene 1	ENSMUSG00000056579	1
Ubap2l	Ubiquitin-associated protein 2-like	ENSMUSG00000042520	4
Ubc	Ubiquitin C	ENSMUSG00000008348	1
Ubl5	Ubiquitin-like 5	ENSMUSG00000084786	2
Uggt1	UDP-glucose glycoprotein glucosyltransferase 1	ENSMUSG00000037470	3
Usp28	Ubiquitin specific peptidase 28	ENSMUSG00000032267	2
Vps13d	Vacuolar protein sorting 13D	ENSMUSG00000020220	1

Wdr43	WD repeat domain 43	ENSMUSG00000041057	1
Wsb1	WD repeat and SOCS box-containing 1	ENSMUSG00000017677	1
Xpo4	Exportin 4	ENSMUSG00000021952	8
Xpo6	Exportin 6	ENSMUSG00000000131	1
Zfp326	Zinc finger protein 326	ENSMUSG00000029290	2
Zfp638	Zinc finger protein 638	ENSMUSG00000030016	9
Zfr	Zinc finger RNA binding protein	ENSMUSG00000022201	4
		**TOTAL SPLICING EVENTS**	**458**

Supplementary Table 4. Analysis of quadriceps fiber area, perimeter and Feret's diameters in VHL and HALO treated delta 7 mice.

	VHL	HALO	Student's t-test
Area (μm2)	177.5 ± 9.653	362.1± 29.49	p= 0.0011
Perimeter (μm)	51.78 ± 1.543	74.22± 2.72	p=0.0003
Min Feret's diameter	12.16 ± 0.23	18.06± 0.75	p=0.0003
Max Feret's diameter	19.31 ± 0.74	27.21± 0.92	p=0.0004

(*p < 0.05; **p < 0.01; ***p < 0.005)

Supplementary Table 5. Analysis of gastrocnemius fiber area, perimeter and Feret's diameters in VHL and HALO treated delta 7 mice.

	VHL	HALO	Student's t-test
Area (μm2)	259.1 ± 57.54	245.3 ± 17.21	p=ns
Perimeter (μm)	60.32 ± 5.95	60.69 ± 2.29	p=ns
Min Feret's diameter	15.07 ± 1.67	15.05 ± 0.47	p=ns
Max Feret's diameter	22.04 ± 2.08	22.82 ± 0.86	p=ns

(ns: not significant)

Supplementary Table 6. List of genes (in alphabetic order) differentially expressed in quadriceps in response to HALO according to RNASeq analysis; used in Supplementary Fig. 6 and 7.

Gene name	Description	Ensembl Id
Ache	Acetylcholinesterase	ENSMUSG00000023328
Adipoq	Adiponectin C1Q and collagen domain containing	ENSMUSG00000022878
Chrna1	Cholinergic receptor nicotinic alpha polypeptide 1 (muscle)	ENSMUSG00000027107
Chrnd	Cholinergic receptor nicotinic delta polypeptide	ENSMUSG0000026251
Dag1	Dystroglycan 1	ENSMUSG00000039952
Egr2	Early growth response 2	ENSMUSG00000037868
Erbb2	Erb-b2 receptor tyrosine kinase 2	ENSMUSG00000062312
Fasn	Fatty acid synthase	ENSMUSG00000025153
Gabrb3	Gamma-aminobutyric acid (GABA) A receptor subunit beta 3	ENSMUSG00000033676
Igf2	Insulin-like growth factor 2	ENSMUSG00000048583
Igf2bp2	Insulin-like growth factor 2 mRNA binding protein 2	ENSMUSG0000033581
Igf2r	Insulin-like growth factor 2 receptor	ENSMUSG00000023830
Itgb3	Integrin beta 3	ENSMUSG00000020689
Kcnj2	Potassium inwardly-rectifying channel subfamily J member 2	ENSMUSG00000041695
Lama2	Laminin alpha 2	ENSMUSG00000019899
Lrp4	Low density lipoprotein receptor-related protein 4	ENSMUSG00000027253
Ntrk2	Neurotrophic tyrosine kinase receptor type 2	ENSMUSG00000055254
Pck1	Phosphoenolpyruvate carboxykinase 1 cytosolic	ENSMUSG00000027513
Postn	Periostin osteoblast specific factor	ENSMUSG00000027750
Scd1	Stearoyl-Coenzyme A desaturase 1	ENSMUSG0000037071
Scd2	Stearoyl-Coenzyme A desaturase 2	ENSMUSG00000025203
Slitrk6	SLIT and NTRK-like family member 6	ENSMUSG0000045871
Srebf1	Sterol regulatory element binding transcription factor 1	ENSMUSG00000020538
Tgfbi	Transforming growth factor beta induced	ENSMUSG00000035493

Legends for Supplementary Data Files 1, 2, and Supplementary Movie 1

Supplementary Data file 1. Complete RNA-seq quantification in spinal cord of delta 7 mice (HALO-treated vs VHL) worksheets: (1) genome-wide expression matrix, (2) differentially expressed genes (DEG) (unadjusted p<0.05), and (3) DEG after multiple testing correction (FDR<0.05).

Supplementary Data file 2. Complete RNA-seq quantification in quadriceps of delta 7 mice (HALO-treated vs VHL) worksheets: (1) genome-wide expression matrix, (2) differentially expressed genes (DEG) (unadjusted p<0.05), and (3) DEG after multiple testing correction (FDR<0.05).

Supplementary Movie 1. Haloperidol treated mice exhibit greater motor strength and activity than VHL. Representative video showing treated SMA mice (P13), highlighting a weaker, tremor-like phenotype in VHL mice compared to HALO.
