# Supplementary material for: Haloperidol induces neuroprotection and enhances neuromuscular function in both murine and human models of spinal muscular atrophy
Source: Exp Mol Med. 2026 Apr 13;58(4):1216–29. doi: 10.1038/s12276-026-01689-0 (PMC13144737; doi:10.1038/s12276-026-01689-0)
Supplement: Supplementary file 2 — Full unedited immunoblotting membranes [file 12276_2026_1689_MOESM2_ESM.pdf]

Supplementary Materials for:

"Haloperidol induces neuroprotection and enhances neuromuscular function in both murine and human models of Spinal Muscular Atrophy"

Giovanna Menduti *et al.*

Corresponding author: Giovanna Menduti, giovanna.menduti@unito.it

This file includes:

- Full unedited immunoblotting membranes

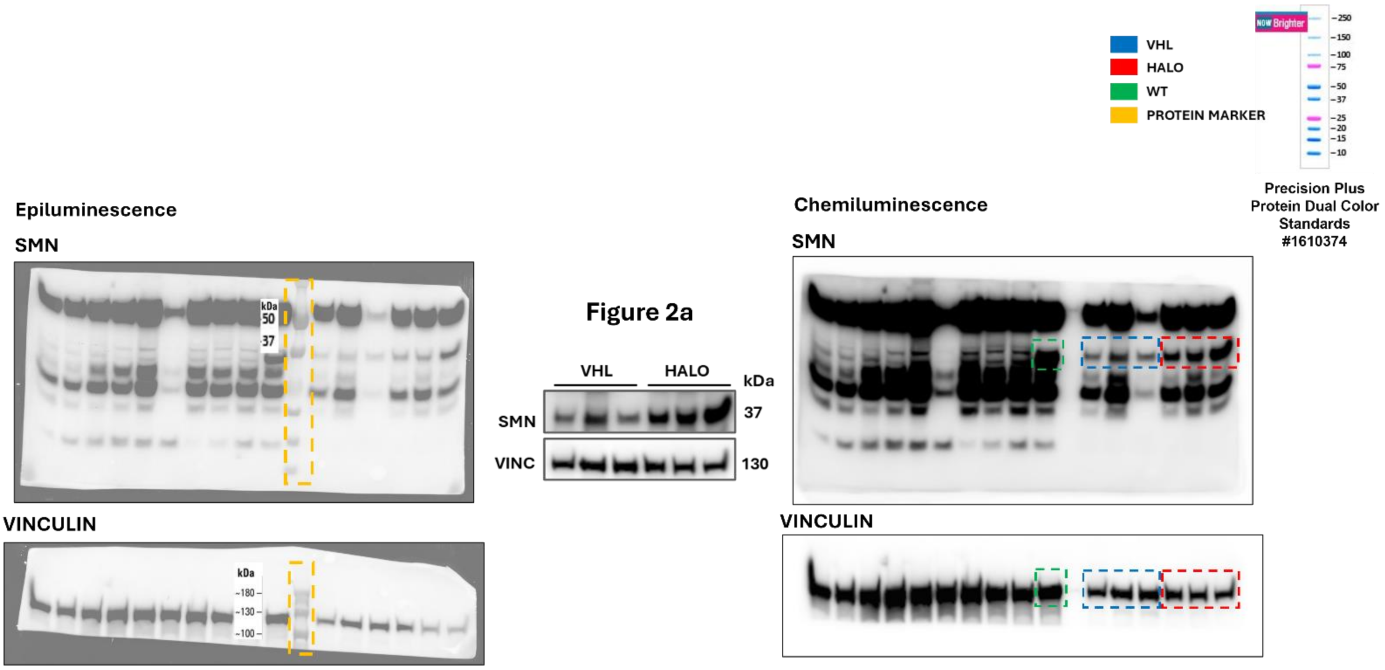

Figure S2

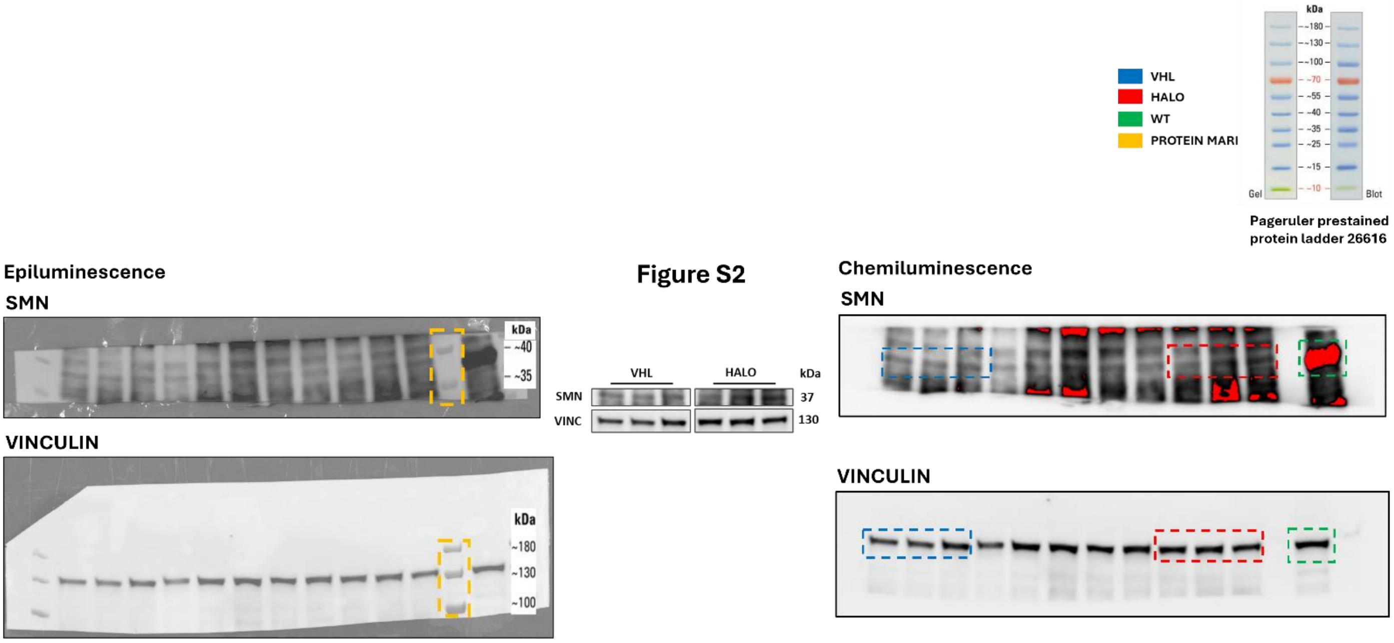

Figure S3

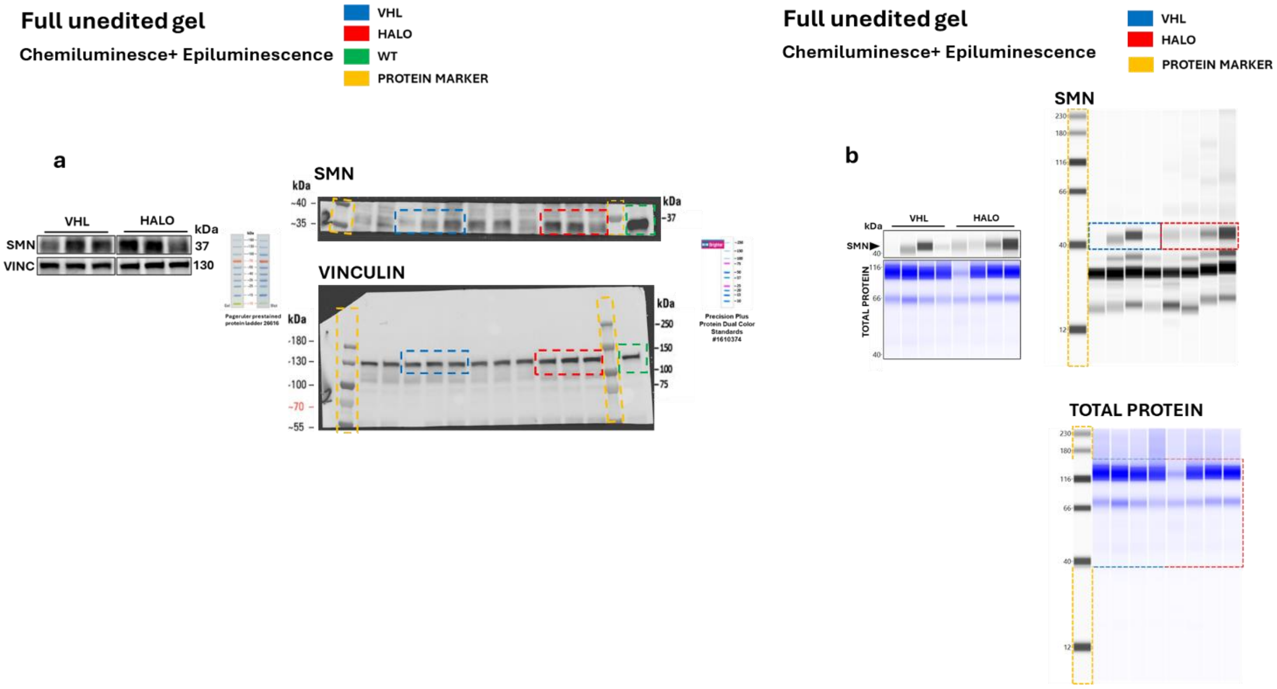

Figure 3e

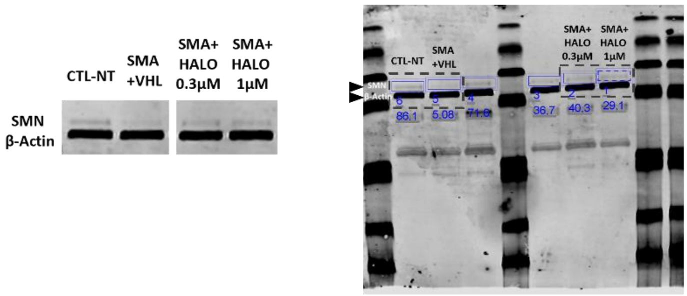

Figure S6d

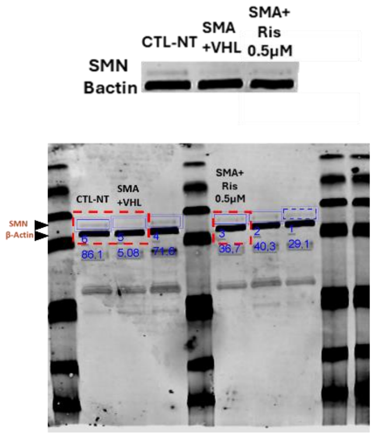

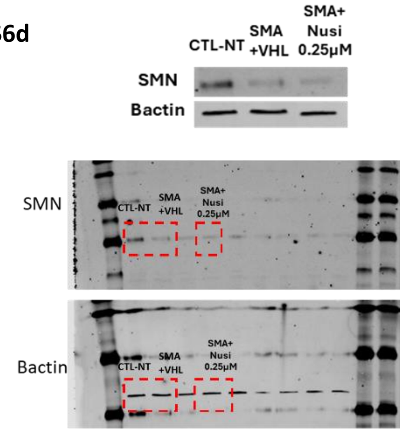

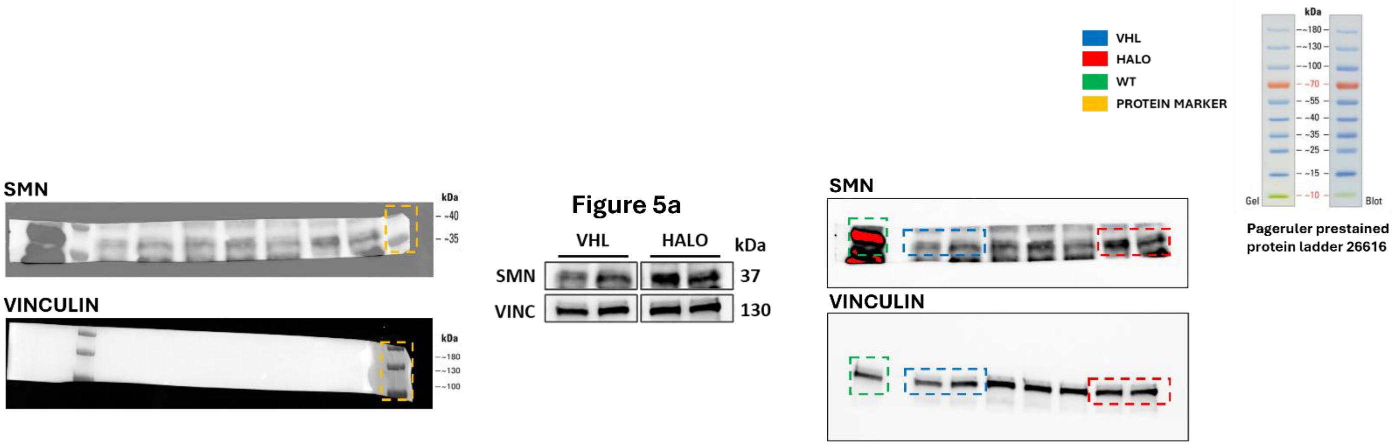
VHL
HALO
WT
PROTEIN MARKER
kDa
~180
~130
~100
~70
~55
~40
~35
~25
~15
~10
Gel
Blot
Pageruler prestained protein ladder 26616
SMN
VINCULIN
Figure 5a
VHL
HALO
kDa
SMN
VINC
37
130

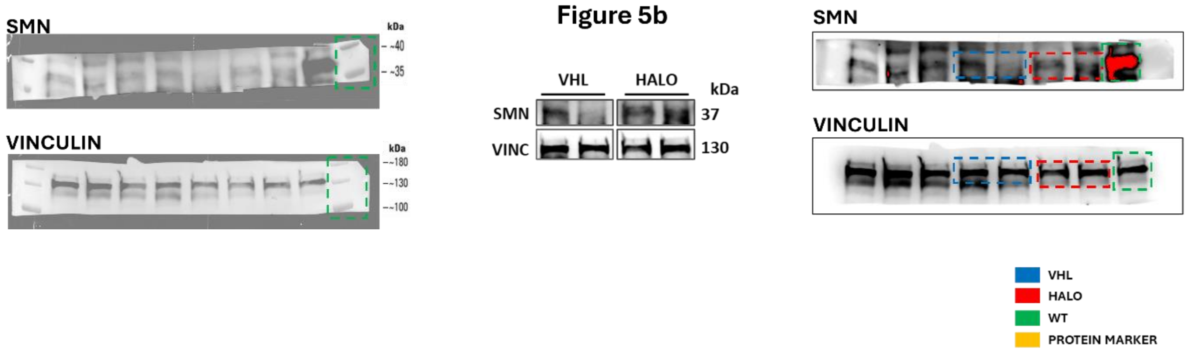
SMN
VINCULIN
kDa
~40
~35
~180
~130
~100
Figure 5b
VHL
HALO
kDa
SMN
VINC
37
130
VHL
HALO
WT
PROTEIN MARKER

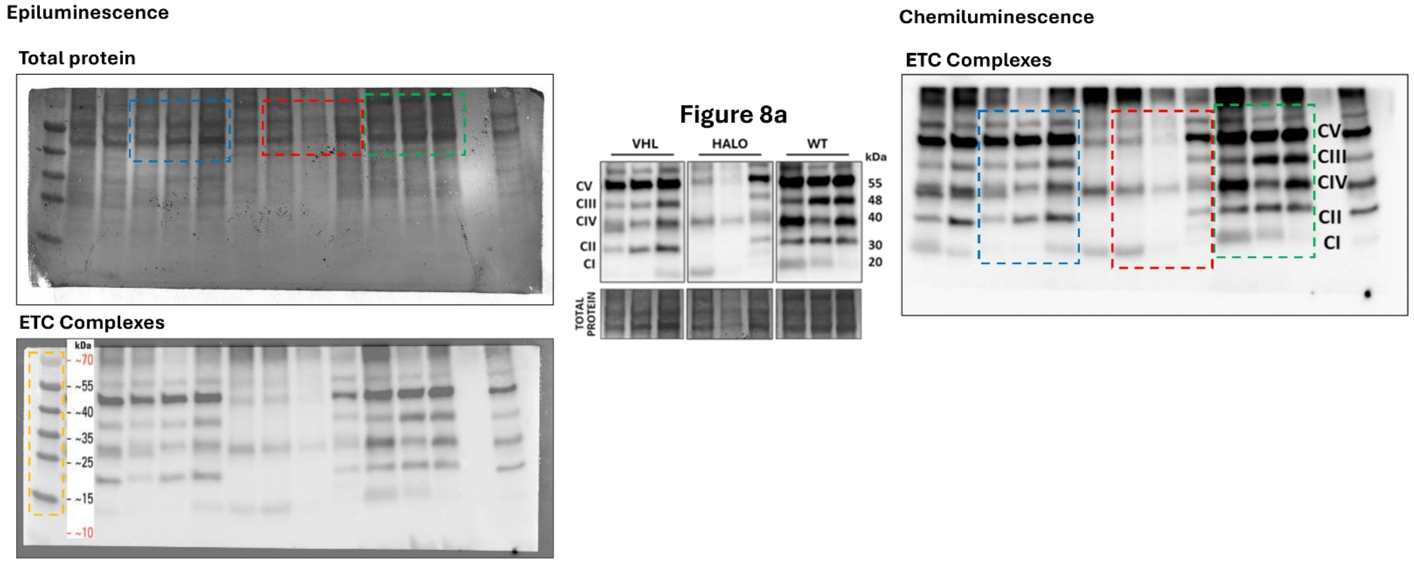
Epiluminescence
Total protein
ETC Complexes
kDa
~70
~55
~40
~35
~25
~15
~10
Figure 8a
VHL
HALO
WT
kDa
CV
CIII
CIV
CII
CI
55
48
40
30
20
TOTAL PROTEIN
Chemiluminescence
ETC Complexes
CV
CIII
CIV
CII
CI

Figure 8c

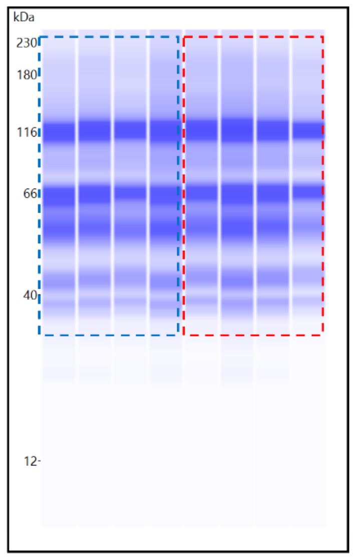

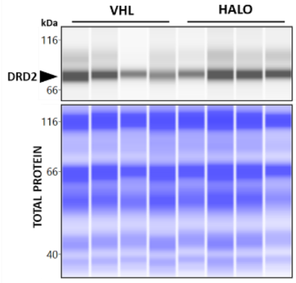

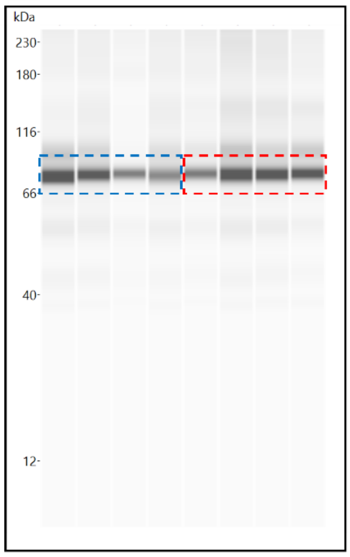

Figure 8d

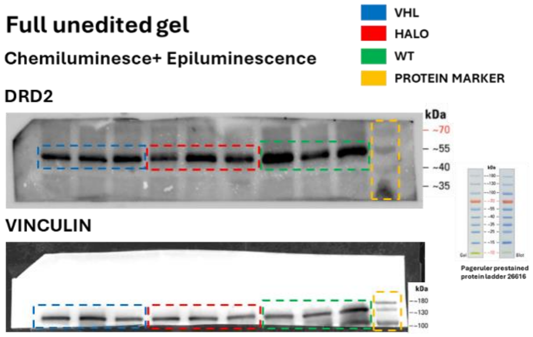

Figure 8e

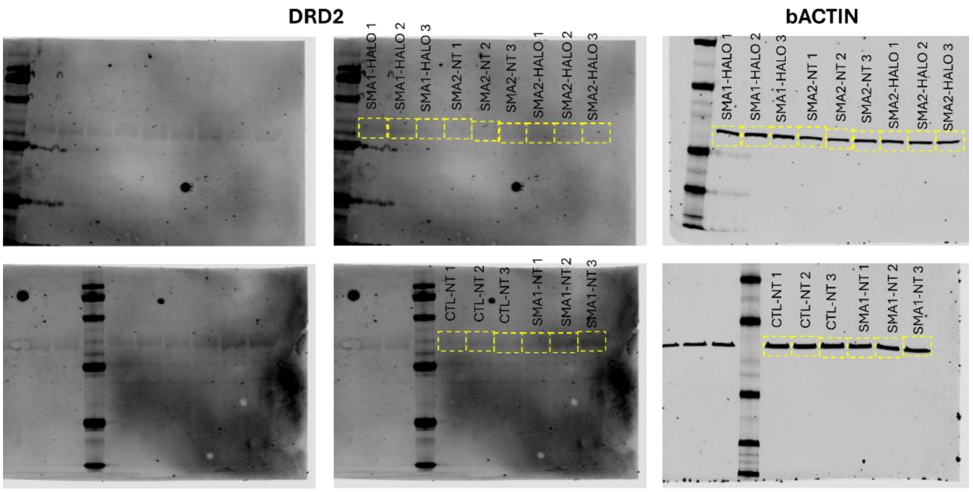

Figure S11

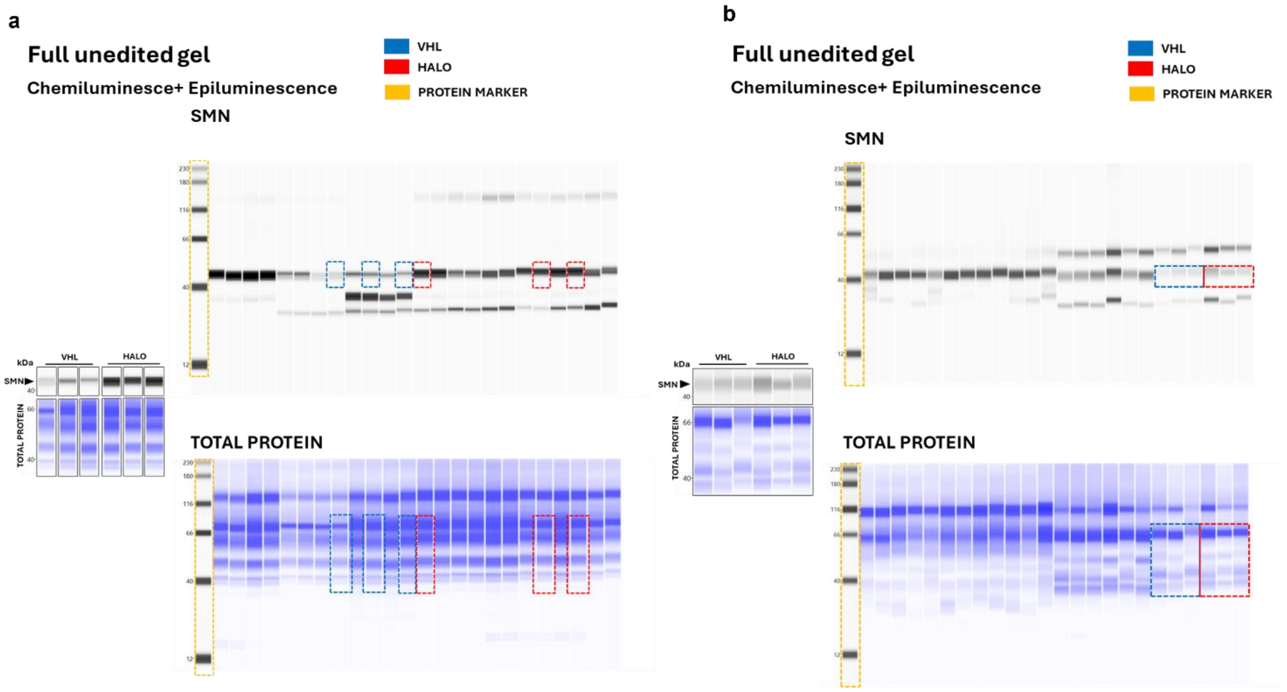
a
Full unedited gel
Chemiluminesce+ Epiluminescence
SMN
VHL
HALO
PROTEIN MARKER
kDa
SMN
TOTAL PROTEIN
TOTAL PROTEIN
b
Full unedited gel
Chemiluminesce+ Epiluminescence
VHL
HALO
PROTEIN MARKER
SMN
kDa
SMN
TOTAL PROTEIN
TOTAL PROTEIN
